# Processing–Microstructure–Properties of Columns in Thermal Barrier Coatings: A Study of Thermo-Chemico-Mechanical Durability

**DOI:** 10.1021/acsami.3c16681

**Published:** 2024-02-13

**Authors:** Siddharth Lokachari, Kah Leng, Acacio Rincon Romero, Nicholas Curry, Gyaneshwara Brewster, Andy Norton, Tanvir Hussain

**Affiliations:** † Centre of Excellence in Coating and Surface Engineering, Faculty of Engineering, University of Nottingham, Nottingham NG7 2RD, U.K.; ‡ Rolls-Royce plc, Derby DE24 8BJ, U.K.; § Rolls-Royce UTC in Manufacturing and On-Wing Technology, Faculty of Engineering, University of Nottingham, Nottingham NG7 2RD, U.K.; ∥ Thermal Spray Innovations, Salzburg 5662, Austria

**Keywords:** thermal barrier coatings, suspension plasma spray, columnar microstructure, CMAS resistance, thermal cycling resistance, erosion resistance

## Abstract

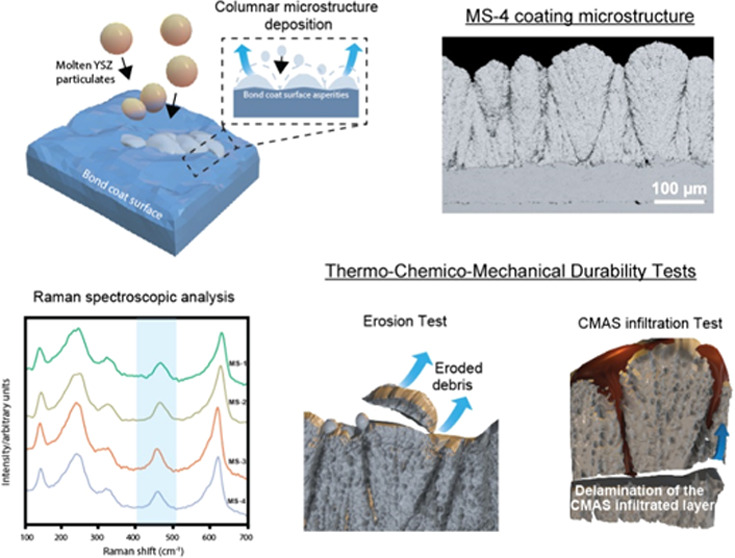

Contemporary gas turbine engines rely on thermal barrier coatings (TBCs), which protect the structural components of the engine against degradation at extremely high operating temperatures (1300–1500 °C). The operational efficiencies of aircraft engines have seen significant improvement in recent years, primarily through the increase in operating temperatures; however, the longevity of TBCs can be potentially impacted by several types of degradation mechanisms. In this comprehensive study, a wide range of novel columnar suspension plasma sprayed (SPS) coatings were developed for their erosion, calcium–magnesium–aluminum-silicate (CMAS), and furnace cycling test (FCT) performance. Through a comprehensive investigation, the first of its kind, we achieved a range of SPS microstructures by modifying the spray parameters and measuring their microhardness, fracture toughness, column densities, and residual stresses using Raman spectroscopy. We were able to produce dendritic, lateral, branched, and columnar microstructures with a unique set of processing parameters. Coatings enhanced with a refined columnar microstructure, achieved by modulating the distance from the plasma torch, exhibited superior thermal cycling resilience. Conversely, the development of a columnar microstructure with dendritic branches, obtained by decreasing the robot’s traversal speed during deposition, bolstered resistance to erosion and minimized damage from molten CMAS infiltration, thereby notably augmenting the coating’s lifespan and robustness. The pursuit of the optimal columnar microstructure led to the conclusion that for each SPS coating, a general framework of optimization needs to be conducted to achieve their desired thermo-chemico-mechanical resistance as the properties required for TBCs are intertwined.

## 1 Introduction

The performance of a gas turbine engine is intricately linked to the turbine entry temperature (TET), where a higher TET is directly proportional to an increased efficiency. With the keen desire for improved performance and power in aircraft engines, there is a continual push toward more arduous operating conditions.^1^ With higher operating temperatures, the bare nickel-based superalloy components may have reached close to their melting temperatures, resulting in the risk of mechanical or environmental failure over an extended period. To overcome this issue, thermal barrier coatings (TBCs) have been employed on these components.^2^ TBCs are thermal insulation layers that protect the underlying metallic substrates from the harsh environment by reducing the surface temperature of the components in the range of 100–300 °C.^3^ A TBC system is typically composed of a substrate, a bond coat, and a ceramic top coat. The bond coat, which is usually made of platinum/nickel aluminide or MCrAlY (M = Co, Ni), is a metallic layer that aims to minimize the thermal strain between the substrate and the top coat and improve the oxidation resistance of the underlying substrate, thereby enhancing the durability of the coating. The ceramic top coat is composed of yttria-stabilized zirconia (YSZ), a highly functional ceramic coating.^4^ The implementation of TBCs on gas turbine engine components has been crucial in improving the lifetime and safety of these components and supports the drive toward a net-zero economy.^5^

**Figure 1:**
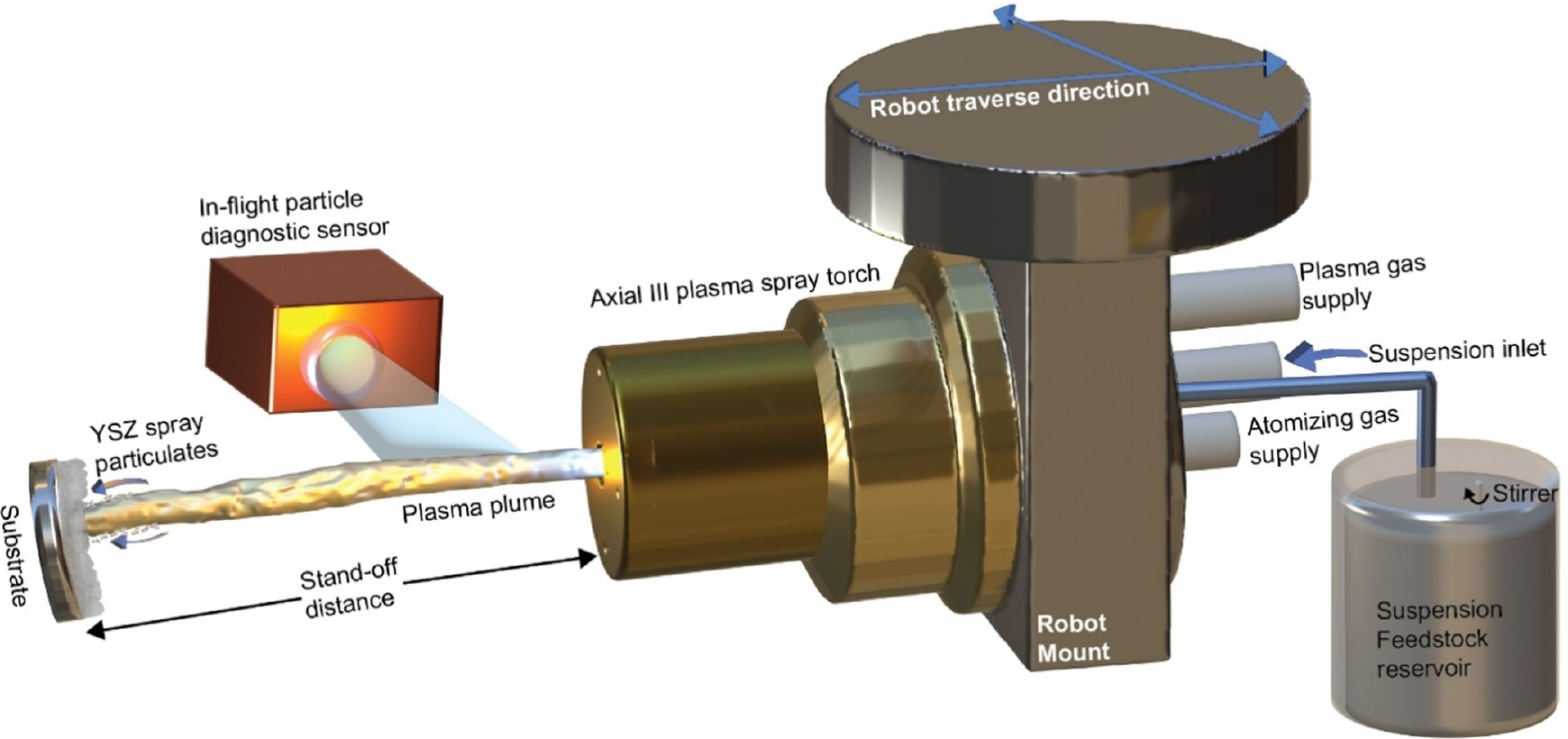
Schematic illustration of an axial suspension plasma spray system.

TBCs are commonly deposited using air plasma spray (APS) or electron beam physical vapor deposition (EB-PVD) methods. The application of TBCs through APS is primarily used for large, stationary components, such as nozzle guide vanes and combustor tiles in aeronautical engines. On the other hand, EB-PVD is utilized for the deposition of TBCs on rotary components such as high-pressure turbine blades. This method is known for its exceptional durability due to its columnar microstructure, which provides strain tolerance and thermal shock resistance. However, it should be noted that the deposition rate of EB-PVD is relatively low.^6^ Additionally, the process requires expensive vacuum chambers and significant installation costs, which can exceed 15 million pounds per unit.^7^ Given the crucial impact of TBCs on efficient air travel, the ingestion of molten environmental siliceous debris with air intake into the combustion chamber of modern jet engine-CMAS (calcium–magnesium–alumino-silicates) has become an ever-burgeoning issue and a pressing priority for research and development.^8−10^ The operating temperatures of the combustion chamber far surpass the melting points (approximately 1300 °C) of the environmental debris.^11,12^ As a result, particles that are ingested undergo a process of liquefaction, which leads to their adherence to the surface of TBCs, potentially compromising their long-term durability.^13^ In the present investigation, an emerging coating deposition process, widely called suspension plasma spray (SPS), was utilized.^14−16^ This technique involves the dispersal of submicrometer or nanosized powder particles within a liquid medium, such as water or alcohol, in the feedstock reservoir to form a colloidal suspension. The suspension is then introduced into a plasma, leading to the atomization of the liquid into fine droplets that contain solid powder particles.^17^ The evaporation of the liquid results in the deposition of fine powder particles onto the substrate, as illustrated in Figure 1.

The spray particulates undergo a complex process of partial or complete melting and sintering, resulting in the formation of agglomerates. An in-flight particle diagnostic sensor is utilized to measure both the temperature and the velocity of the spray particulates. Upon impact of the droplets on the substrate, a finely structured coating is deposited. Over the past decade, a significant body of work was devoted to studying the performance (CMAS and FCT) of SPS 7-8YSZ coatings.^18−20^ However, within this vast literature, a gap exists that can correlate the fundamental performance of these coatings with their associated columnar architecture. There is no comprehensive paper in the literature that can shed light on the performance of these coatings and tie those back to their microstructure.

The utilization of SPS for TBCs is influenced by its ability to generate coatings with a columnar microstructure,^19^ similar to that of EB-PVD coatings. This microstructural attribute is highly sought after in the gas turbine industry due to the strain-resistant nature of these coatings, resulting in increased longevity. The SPS coatings exhibit versatility in meeting the demands of various industrial domains spanning from aeronautics to the endoprosthesis industries, with a significant emphasis on gas turbine engine coatings. These engines represent a pivotal element in the propulsion systems of both civilian and defense aircraft, supporting a vast array of aviation operations. The overall worth of airlines’ production is estimated to be $2.94 trillion in 2022, underscoring the tremendous economic significance of this industry.^20^ In this study, we aimed to conduct a comprehensive analysis of the process parameter window of the plasma spray suspension and the effect it has on the development of columnar microstructure coatings. The purpose was to gain a better understanding of the critical parameters that influence the formation of the columns and to investigate how these microstructures influence thermo-chemico-mechanical performance.

## 2 Experimental Methods

### 2.1 Materials

To realize columnar microstructured coatings, commercially available 8 wt % YSZ suspended in ethanol, with a solid loading of 25 wt %, was utilized. The suspension was supplied by Treibacher Industrie AG (Althofen, Austria). The Backscattered Electron Image (BSE) of the YSZ particulates is shown in Figure S1, and the median particle size distribution of D50 was 500 nm. The suspension was homogenized at 50 rpm (Capco ball mill, Suffolk, U.K.) for 1 h prior to the deposition process to mix the YSZ particles uniformly and mitigate the sedimentation of agglomerated particles adhered to the bottom of the container.

### 2.2 Coating Deposition

The coatings were deposited onto Inconel 718 superalloy substrates with a thickness of 3 mm and a diameter of 12.7 mm. The surface of the substrates was first roughened through a grit blasting process using a Guyson grit blaster prior to coating. The pressure during this process was 6 bar, and the grit media used was F100 brown alumina, with a size range of 0.125–0.149 mm. After grit blasting, the substrates were cleaned in an ultrasonic bath with industrial methylated spirit (IMS) for approximately 5 min to remove any contaminants on the surface. The next step in the coating process was the deposition of the cobalt-based bond coat, MCrAlY (CoNiCrAlY) powder, using a high-velocity oxygen-fuel (HVOF) spray process with a commercial MetJet IV spray torch (Metallisation Ltd., Dudley, U.K.). The powder used was CO-210-24, obtained from Praxair in Germany. A detailed description of the bond coat deposition is published in refs (21,22). Finally, the top coat was deposited using a tri-anode/tri-cathode high-power DC plasma torch system-Axial III (Northwest Mettech Corp.). An exit nozzle diameter of 0.375 in. and a suspension injector of 250 μm were used for the spray campaign. The suspension feedstock was fed axially into the plasma plume using a Mettech NanoFeed 350 suspension feeder. The number of robotic passes was maintained at a constant of 40 for all deposition conditions. Through the refinement of spray parameters, including the precise modulation of individual carrier gas composition, total gas flow rate, robot raster speed, and manipulation of the spray torch stand-off distance, it was possible to develop columnar microstructured coatings. The coating deposition is mainly dependent on the heating of spray particles (PT) in-flight and their velocity of impact (PV).^23^ The velocity and spatial distribution of the hot spray particulates are measured by a commercially available sensor—Accuraspray 4.0 (Tecnar, Quebec, Canada), which employs CCD cameras by which the velocity is deducted from the traces of spray particulates within an exposure time range (from 1 to 10 μs).

### 2.3 Microstructural Characterization

The surface roughness (*S*_a_) of the as-deposited coatings was measured using infinite focus Alicona G5+ (Bruker, Switzerland); ten different measurements were obtained at the center and edges on either side. The cross-sectional microstructures of coated samples were thoroughly characterized using a variety of techniques. The samples were evaluated in both their as-deposited state and in states of degradation caused by different regimes of exposure. To obtain cross-sectional samples, the coated samples were cut at their geometric center using a precision cutting wheel saw (MetPrep, Coventry, U.K.) at a relatively slow speed to minimize damage caused by the cutting process. The resulting cross-sectional samples were mounted with epoxy resin and hardener (Struers, Rotherham, U.K.) and then ground sequentially using coarse (#500) to fine grit SiC abrasive papers (#2500) (MetPrep, Coventry, U.K.) before being polished to a surface finish of 1 μm using diamond polishing.

The cross-sectional microstructures and surface topography were examined by using a scanning electron microscope (FEI Quanta 600) in both secondary electron (SE) and backscattered electron (BSE) modes. A spot size of 50–60 nm, a working distance of 9 mm, and an acceleration voltage of 20 kV were used as imaging parameters. The ImageJ analysis suite (NIH) was used to measure the thickness of the coating, and the reported data represent the average and standard error of the coating thickness using secondary electron images (×300) covering 1 cm of the coating cross-section with 5 images. X-ray diffraction (XRD) analysis was performed using a D8 Advance DaVinci system (Figure S2). The diffractograms were obtained with Cu–Kα radiation with a wavelength of 1.54 Å in Bragg–Brentano configuration in the range from 10° to 90° 2°, using a 0.02° step size and 0.15 s of counting time in each step. The phase identification was performed using the Diffract.suite eva software (Bruker, Coventry, U.K.).

### 2.4 Mechanical Properties

The microhardness of the coating was measured utilizing a Vickers microhardness indenter in conjunction with an optical microscope (manufactured by Buehler). The test was performed on polished cross sections, wherein a load of 50 gf (0.5 N) was applied for 10 s, resulting in the creation of five independent indentations centered on the coatings and oriented parallel to the substrate. The in-plane hardness of the coatings was measured with a load of 50 gf by placing five distinct indentations on the polished topographical surface of the coating, which was obtained perpendicularly at incremental distances of 25, 50, 100, and 150 μm from the coating–substrate interface. The resultant microhardness value was recorded as an average with its standard error in the mean. Furthermore, the fracture toughness of the coatings was determined by the cracks induced by indentations applied with a significantly higher load of 500 g of friction (5 N). The fracture toughness of the Thermal Barrier Coating (TBC) was then calculated using eq 1(24)
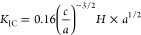
1Where “*K*_IC_” is the fracture toughness (MPa·m^1/2^), “*H*” is the indentation microhardness (MPa), “*a*” is the indent half-diagonal length (m), and “*c*” is the average crack length measured from the center of the indent to the tip of the crack (m). The length of the cracks must satisfy the median crack criterion *c*/*a* ≥ 2.5 for the validity of the measurements.^24^

### 2.5 Furnace Cycling Test (FCT)

The furnace cyclic tests (FCT) were conducted three times using the same batch of coatings deposited during the same campaign. This approach was taken to maintain uniformity and consistency across the tests. A programmable bottom-loading isothermal furnace (CM Furnaces Inc., Bloomfield) was used for this test, and each cycle of the test consisted of three stages: heating, dwelling, and cooling. The heating stage involved increasing the temperature of the sample to 1135 °C within a period of 10 min. The sample was then held at this temperature for 45 min during the dwelling stage. Finally, in the cooling stage, forced air cooling was used to bring the temperature of the sample below 100 °C within approximately 30 min. The extent of spallation was monitored using a high-definition Webcam (Logitech C930e, Lausanne, Switzerland) that recorded an image at progressive 1-min intervals. The test continued until the emergence of a spallation area exceeding 20% of the top coat’s surface.

### 2.6 Erosion Test

The erosion test was performed following the ASTM G76 standard on as-deposited SPS coatings using a modified Portablast sandblast unit by SJS Engineering in Nottingham, U.K., at room temperature. For the erosion test, all of the coatings were deposited with the objective of reaching a consistent thickness of 300 μm, ensuring uniformity throughout the test. Prior to the measurement of their initial mass using a precision balance, the TBC samples were cleaned using an ultrasonic bath with ethanol and then dried. The erosion test utilized 220# white fused alumina grit with an impact rate of 5 g/s at an angle of 90° and an average erodent pressure of 0.1 MPa. The stand-off distance between the substrate and erosion tester was set to 100 mm. After each test, the TBC sample was cleaned and dried to measure the mass loss due to erosion. Post-erosion analysis included examining the eroded regions on the sample surface and in cross-section with scanning electron microscopy (SEM), following the previously described sample preparation procedure. The erosion rate (*g*/*g*), denoting the proportion of mass depletion in TBCs (*g*) relative to the mass of erodent (*g*) employed to induce erosion, was computed.

### 2.7 Raman Spectroscopic Analysis

The LabRAM HR spectrometer (Horiba Jobin YVON, Japan) was modified with the addition of an automated xyz stage (Marzhauser, Germany) to perform Raman spectroscopy. Prior to collecting spectra, the instrument was calibrated using a standard Si (100) reference band at 520.7 cm^–1^. Spectra were obtained for cross-sectional analysis with a green laser with a wavelength of 532 nm. A 300 μm pinhole and 100× objective were also used. Two different wavelengths were utilized to avoid the fluorescence effect. Signals were detected with a Synapse detector (Horiba, Japan) to create spectra. Each individual spectrum was collected for 20 s and repeated 3 times to eliminate artifacts (cosmic spikes) generated by the cosmic rays to improve the signal-to-noise ratio. Spectra were corrected by applying linear baseline subtraction to eliminate any residual fluorescence using Labspec 6 software (Horiba Jobin YVON, Japan) and normalized. The Raman spectra of tetragonal zirconia were analyzed and fitted using a Breit–Wigner profile, which is an asymmetric Lorentzian function. The analysis was performed using the OriginPro package (OriginLab Corp., Northampton).

The residual stresses in YSZ TBCs are typically deemed to be the primary contributor to the eventual failure.^25,26^ This study employs the Raman method of as-deposited coatings to gauge the stress in the YSZ top coat, utilizing the same methodology as Limarga et al.^27,28^ The in-plane residual stresses (σ_in-plane_) are calculated by calculating the Raman peak shift Δν, as per eq 2
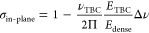
2Where Δν is the Raman peak shift from the reference as-deposited state, ν_TBC_ is the Poisson’s ratio of the YSZ, TBC is 0.25,^29^ and Π is the piezo spectroscopic coefficient and pertains specifically to the dense 7YSZ under uniaxial stress. For a frequency of 465 cm^–1^, the coefficient measures 2.01 cm^–1^/GPa.^30^*E*_TBC_ and *E*_dense_, respectively, denote the in-plane Young’s modulus of the top coat of SPS and the fully dense YSZ. The former is estimated to be around 30 GPa through^31^ a three-point bending test, whereas the latter has been reported to have a Young’s modulus of approximately 210 GPa.^27^

### 2.8 CMAS Exposure

The CMAS solution was created by mixing CMAS powder (Oerlikon Metco, Cheshire, U.K.) with a nominal composition of 35CaO-10MgO-7Al_2_O_3_-48SiO_2_ in mol % water and deionized (DI) water in a 1:9 ratio. The CMAS was then evenly distributed on various types of TBCs using an airbrush kit. The solution was constantly agitated using a magnetic stirrer on an Isotemp hot plate (Fisher Scientific, Loughborough, U.K.). In adherence to established guidelines within the high-temperature community, a concentration of 15 mg/cm^2^ for CMAS was selected.^32^ Following the deposition of CMAS, the specimen was subjected to a hot plate and gradually heated to an approximate temperature of 100 °C to facilitate the evaporation of DI water that was intermixed with the CMAS solution. Pre- and postdeposition weight measurements of the sample were recorded, and this iterative process was repeated until the desired concentration level was attained. The CMAS test was conducted in a box furnace (Elite Thermal Systems Ltd., Leicester, U.K.). All samples were heat-treated at 1250 °C for 5 min at a ramp rate of 10 °C/min. The furnace was set to cool to 700 °C at the same ramp rate, 10 °C/min, and then a slower ramp rate (5 °C/min) was used to cool to room temperature to reduce thermal shock behavior that may occur in the glassy phase.

## 3 Results

### 3.1 Process Parameter Window Development

In this study, significant factors that impact the plasma drag forces, which are crucial to the formation of columnar microstructures, were meticulously selected. Our starting spray parameters were a pre-established set of reference parameters from the equipment manufacturer and suspension provider, labeled reference parameter (MS-1). This has been extensively reported as the go-to process parameter for SPS YSZ coatings.^33,34^ These factors include the suspension feed rate (MS-2), which is the rate at which the suspension material is introduced into the nozzle, stand-off distance (MS-3), which pertains to the spatial separation between the spray torch and target surface, and robot speed (MS-4), which is the traversing rate at which the robot carrying the spray torch rasters with respect to the target surface. This investigation delves into the interplay of three elements and their impact on the final outcome of the spray process aimed at enhancing the columnar microstructure. Table 1 enumerates the spray parameters utilized in this study, with the reference parameter generating a particle temperature of 2950 °C and a velocity of 600 m/s. The reduction in MS-3 elevates the drag forces, leading to a higher particle velocity of 678.3 m/s and a higher deposition rate than that of the MS-1 coating, owing to the limited time for deceleration before adherence onto the substrate. While the MS-2 and MS-4 coatings possess particle temperature and velocity values similar to those of the MS-1 coating, the MS-4 coating demonstrated the highest deposition rate among all of the coatings.

**Table 1:** Axial III Spray Parameters

spray parameter variation	stand-off distance (mm)	robot speed (mm/s)	feed rate (mL/min)	thickness (μm)	deposition rate (μm/pass)
Microstructure-1 (MS-1)	100	1600	100	231 ± 4	5.8
Microstructure-2 (MS-2)	100	1600	**45**	126 ± 4	3.2
Microstructure-3 (MS-3)	**75**	1600	100	340 ± 5	8.5
Microstructure-4 (MS-4)	100	**1000**	100	392 ± 5	9.8

### 3.2 Microstructural Characterization and Surface Attributes of the Coatings

**Figure 2:**
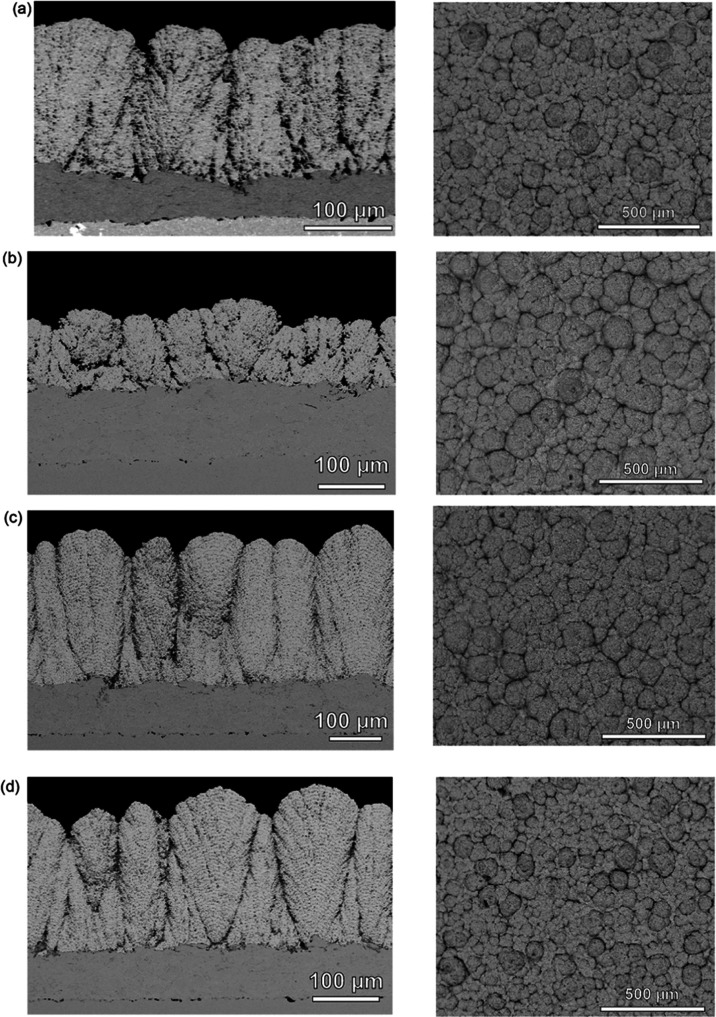
Cross-section (left) and topographical (right) backscattered electron (BSE) images of the coatings: (a) reference: MS-1, (b) change in the suspension feed rate: MS-2, (c) decrease in the stand-off distance: MS-3, and (d) reduction in the robot raster speed: MS-4.

The surface topographical BSE images (Figure 2) revealed that the coatings’ microstructure displayed a rudimentary columnar arrangement characterized by clusters that resembled cauliflower terminals, along with merged microfractal structures.

The microstructure of MS-2 coatings (Figure 2b) was distinct with unique stacking of columnar lamellae compared to MS-1 coating (Figure 2a), also evident from the topographical BSE images, suggesting that the low suspension feed loading is responsible for this unique stacking. Figure 2c,d demonstrates that the reduction in the stand-off distance and robot traverse speed, critical ancillary parameters of the system, significantly affect the columnar features of these coatings, which are characterized by topographical capped cupola structures extending from the bottom to the top of the coating. In the forthcoming sections, we will delve into the broader implications of the impact of process parameters and their interactions on the thermo-chemico-mechanical degradation of these coatings.

**Figure 3:**
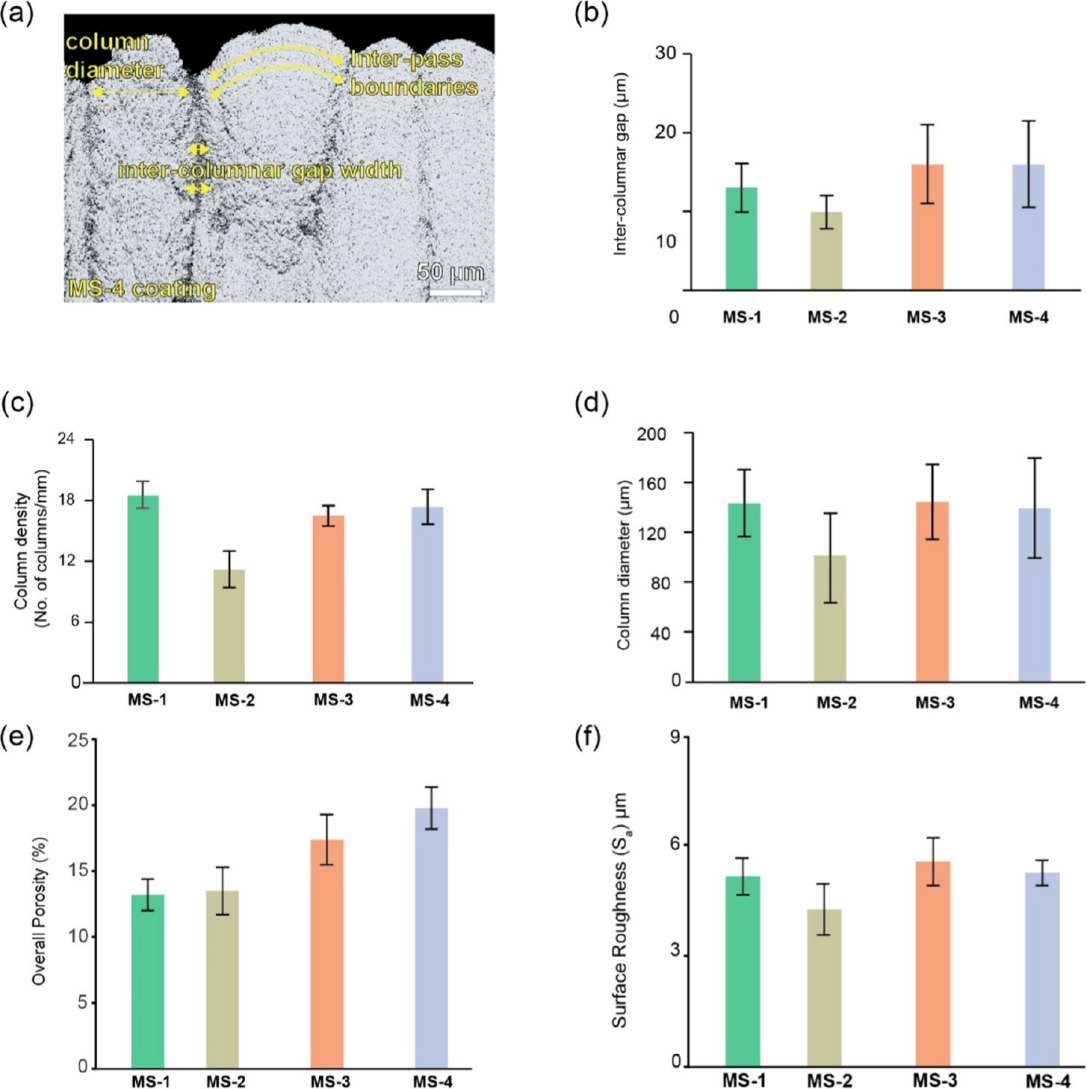
(a) Characterization of the columnar features using a backscattered electron (BSE) image of MS-4 coating, (b) intercolumnar gap width of the coatings, (c) column density of the coatings, (d) column diameter of the coatings, (e) overall porosity, and (f) surface roughness of the coatings.

The characterization of the coating was performed with regard to its distinctive columnar features, including intercolumnar gaps, column density, interpass porosity bands (IPBs), and column diameter (Figure 3a). These individual features play a crucial role in determining the overall porosity of the coating. Intercolumnar gaps or vertical cracks developed perpendicularly between two adjacent columns distinguish the columns generated by SPS and EB-PVD processes.^35,36^ Coatings exhibit porosity that is prominently visible in bandlike formations developed parallelly as a result of the transition between each raster of the spray torch during the deposition.

The formation of IPBs results from airborne YSZ particles, which tend to travel predominantly along the outer regions of the plume rather than through the central core of the plasma. Within this specific area, the particles undergo a decrease in speed and temperature, which can result in their partial melting or resolidification upon deposition. Consequently, these conditions contribute to the overall porosity. MS-1, MS-3, and MS-4 coatings exhibited similar columnar features, as shown in Figure 3b–d. The MS-2 coating indicated wider coalesced columns, leading to a decrease in column density along the cross-section compared to MS-1, MS-3, and MS-4 coatings. The column diameters are presented in Figure 3c. It was noted that the column diameters of the coatings were more than twice that of EB-PVD, which are typically in the range of 15 – 30 μm.^37,38^ The overall porosity of the coatings is presented in Figure 3e, with due consideration of fine, coarse, and interporosity bands that are inherent microstructural features of each coating. MS-4 coating had relatively high overall porosity compared to other coatings, 19.8 ± 1.6%, due to the presence of more intercolumnar gaps and microscale porosities, resulting in the highest material deposition rate, as seen in Table 1. The surface roughness of the coatings is presented in Figure 3f. MS-2 coatings exhibited relatively less surface roughness compared to MS-1, MS-3, and MS-4 coatings.

### 3.3 Impact of Porosity on Microhardness of the Coatings

**Figure 4:**
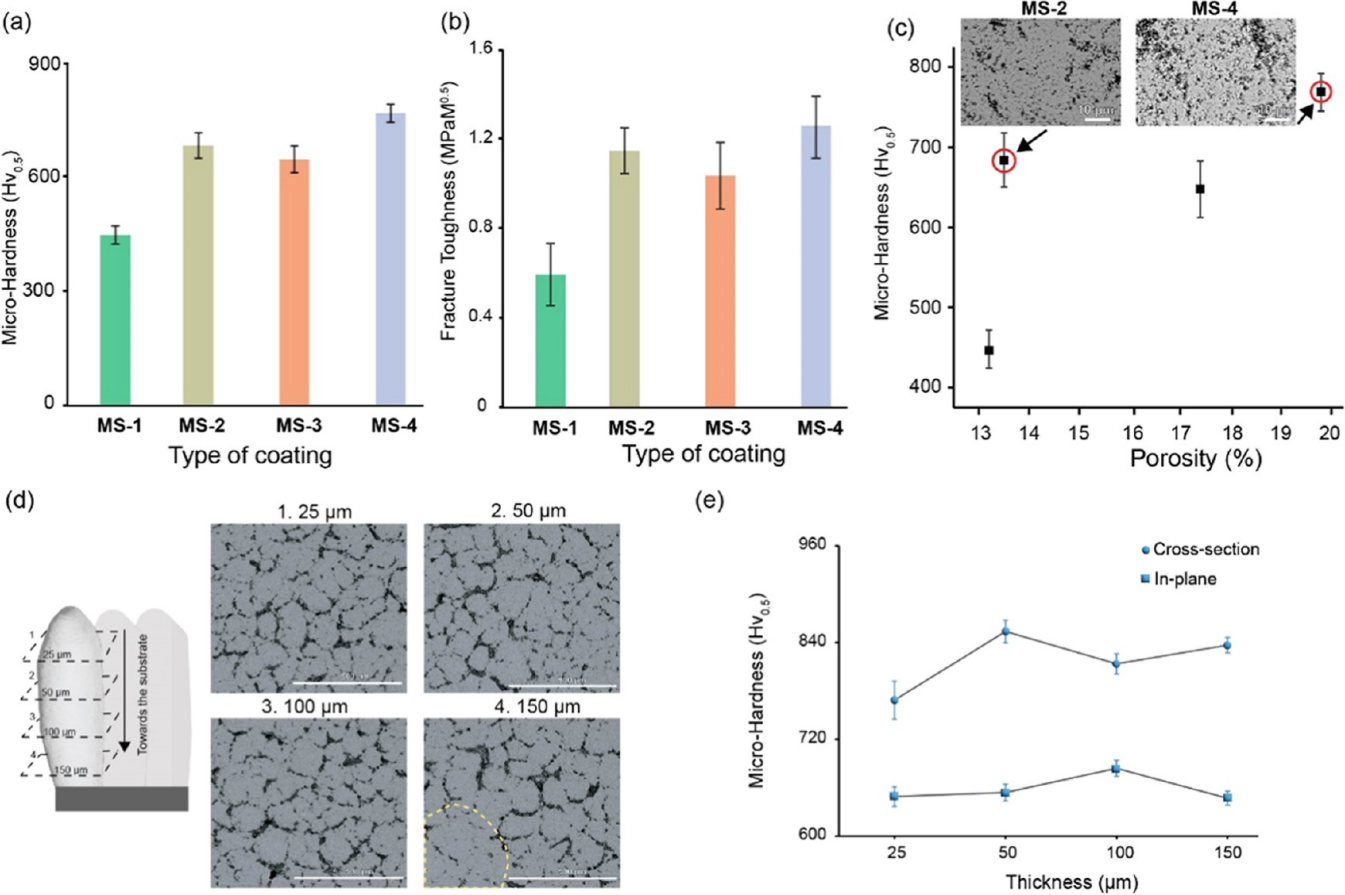
(a) Vickers microhardness, (b) fracture toughness, (c) variation of coating microhardness versus porosity (%), (d) periodical topographic BSE images of MS-4 coating from 25–200 μm toward the substrate, and (e) in-planar vickers microhardness measured on topography: comparison between the cross-section and in-planar microhardness of the MS-4 coating.

The microhardness values of the coatings are listed in Figure 4a. The microhardness of the MS-1 coating was found to be 447 ± 23 Hv_0.5_, and the rest of the coatings exhibited higher hardness values compared to that of the MS-1 coating. MS-4 coating exhibited the highest microhardness of 758 ± 20 Hv_0.5_. The fracture toughness (*K*_IC_) of the coatings was calculated using eq 1, as displayed in Figure 4b, which followed a trend similar to the microhardness values. MS-4 coating had the highest fracture toughness (1.33 ± 0.12 MPa·m^0.5^) among the coatings, and the MS-1 coatings had the least fracture toughness (0.62 ± 0.1 MPa·m^0.5^). The indent impressions of the coatings are represented in Figure S3, and it is evident that the in-plane cracks (parallel to the substrate) were more dominant than the out-of-plane cracks (perpendicular to the substrate) in all of the coatings. This anisotropic behavior suggests that the coatings are more vulnerable to in-plane cracking. Previous research regarding the relationship between porosity and microhardness revealed that less porous SPS coatings exhibited a higher microhardness. The BSE images with high magnification of the MS-2 coating in Figure 4c reveal a dense structure with relatively lower porosity, resulting in higher microhardness. Conversely, the MS-4 coating displays a highly porous nature with the highest microhardness. The increased porosity of the MS-4 coating is linked to the existence of molten/semimolten submicron particulates (∼ 800 nm) within the IPBs, as depicted in Figures 4c and S4.

To delve deeper into understanding the fracture toughness and microhardness properties of the MS-4 coating, in-planar microhardness was performed on the coating topography, as shown in schematic Figure 4d. The BSE images at incremental layers at 25, 50, 100, and 150 μm in Figure 4d indicate that the columns generated in the coating were internally dense, with intercolumnar spacing separating individual columns along the diameter. From the topographical image at 150 μm, a hindsight view of the clustering of columns was evident, which sheds light on the segregated growth into individual columns at 100 μm that arose from the cluster. There was no significant difference in the incremental layers in-planar microhardness values of the MS-4 coating, as shown in Figure 4e; this might be attributed to the uniform melting of the spray particulates, and the values were in agreement with the columnar coatings obtained by Curry et al.^39^

### 3.4 Characterization of the Residual Stress Signatures Using Raman Spectroscopy

Residual stresses are the driving force for TBC failure and the origin of microcracks leading to a detrimental failure.^40,41^ Raman spectroscopic analysis of the residual stresses present in the coatings postdeposition shed light on their durability as we progress to the thermo-mechanical analysis. To understand the influence of the residual stresses on the coating failure, the Raman peak shifts were analyzed across the as-deposited cross-section of the coating.

**Figure 5:**
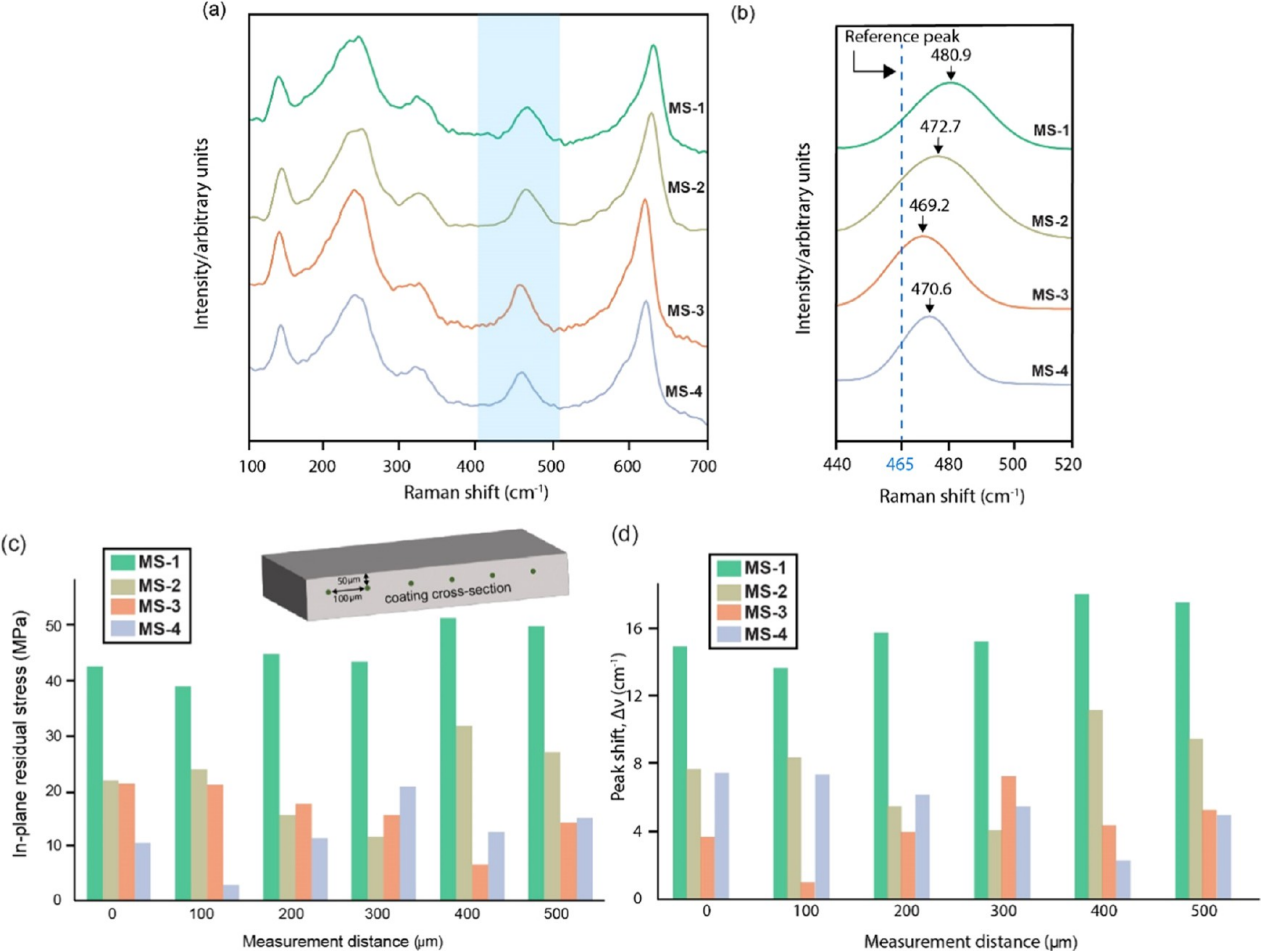
(a) Average Raman spectra across the cross-section of the coatings, (b) the average Breit–Wigner Lorentzian fitting of the individual stress peaks and comparison with the reference stress peak at 465 cm^–1^, and (c), (d) the in-plane residual stresses plotted across the coatings’ cross-section.

Figure 5a represents the average spectra of the coatings and the corresponding stress-sensitive peak shifts in the range of 440–520 cm^–1^ fitted in Figure 5b. The MS-1 coating revealed the maximum in-plane residual stress of 51.7 MPa located at 400 μm post coating deposition, and the MS-4 coating possessed the least residual stress value of 5.5 MPa located at 100 μm. However, the MS-3 coating exhibited consistent minimal residual stress values and corresponding peak shifts throughout the cross-section of the coating, as shown in Figure 5c,d. The average in-plane residual stress for the MS-1 coating was 45.4 ± 4.3 MPa, while the MS-3 coating was 12.3 ± 5 MPa, as shown in Figure S5.

### 3.5 Role of Fracture Toughness on the Erosion of Coatings

**Figure 6:**
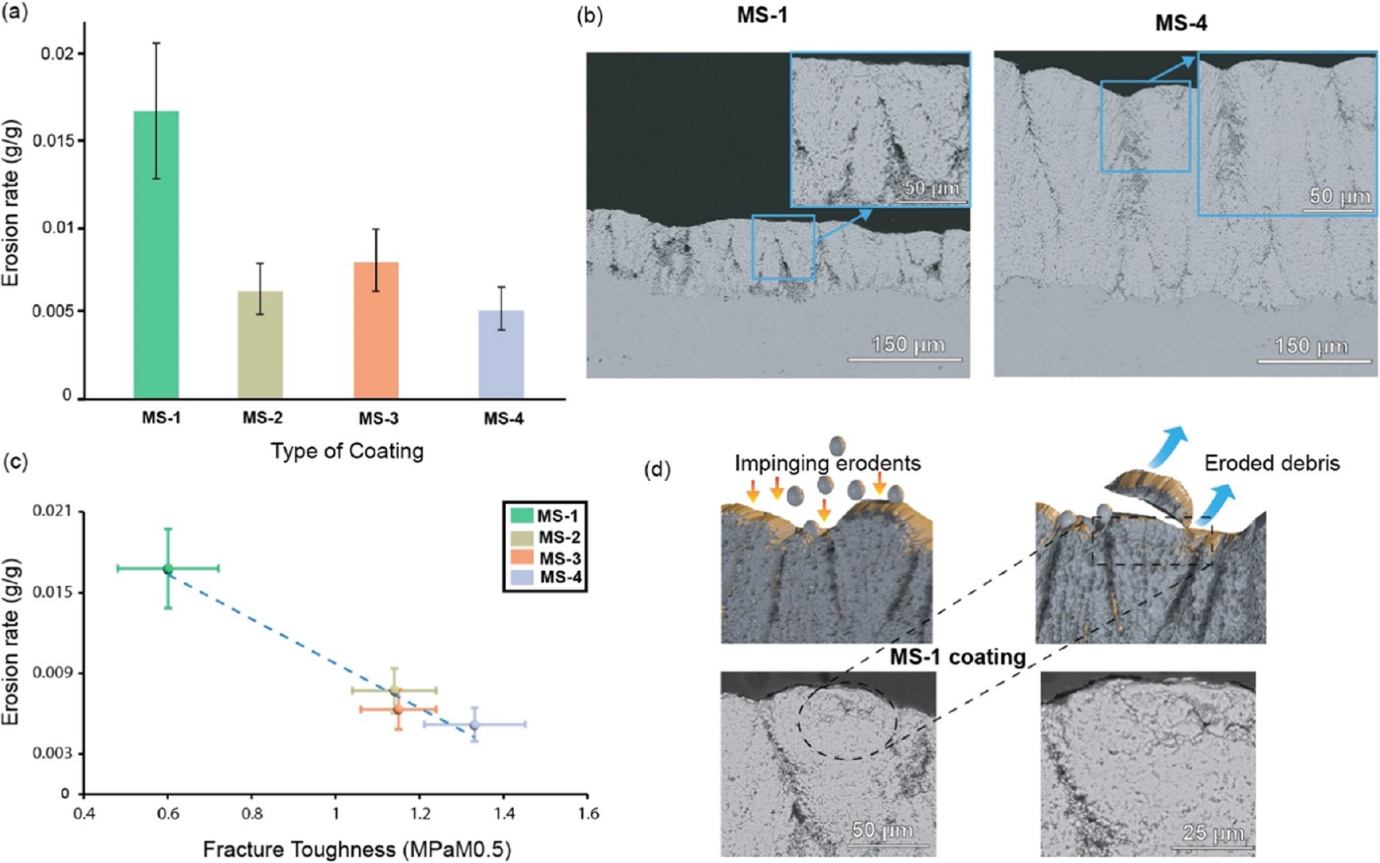
(a) Erosion rates of coatings at an impact angle of 90° for a duration of 25 s, (b) cross-sectional BSE images of the MS-1 and MS-4 coatings, (c) the change in the erosion rate with respect to the Fracture toughness of the coatings, and (d) illustration of the tunneling mechanism involved in the erosion of top coat by impinging erodents.

The erosion rate of the coatings is shown in Figure 6. Impaction of the erodent involves the maximum transfer of energy in comparison with other inclined impact angles, as studied by other researchers.^42,43^

The coatings were ranked similarly to their mechanical properties evaluated by the indentation technique. MS-4 coating showed the lowest erosion rate among the coatings, with a thickness loss of 60 μm and mass loss of 30 mg. Cross-sectional BSE images of the MS-1 and MS-4 coatings are shown in Figure 6 to cover two extreme ranges of erosion. It was observed that the MS-1 coating did not retain any columnar features post erosion. A higher magnification image of the MS-1 coating showed microcracks emanating from the top layer, which is indicative of a tunneling mechanism. The MS-1 coating was subjected to the highest erosion, with a thickness loss of 220 μm and mass loss of 80 mg, majorly due to their poor fracture toughness. The MS-2 coating was ranked next to the MS-4 coating, exhibiting good erosion resistance with a thickness loss of 90 μm and a mass loss of 40 mg. This might be attributed to lesser intercolumnar spacing and better fracture toughness properties compared with MS-1 and MS-3 coatings. Both MS-4 and MS-3 coatings performed well in terms of the erosion test, with MS-4 coatings exhibiting marginally better erosion resistance. It was observed that the columnar features of MS-1 coating were decimated due to the alumina erodents, as illustrated in Figure 6d, thereby generating lateral cracks in the cupola region of the coating, as shown in the magnified BSE images (Figure 6d). Wellman et al. reported that the presence of coarse porosities favored maximum material loss, which favored crack initiations in EB-PVD coatings.^38^ This phenomenon is also valid for the coarse porosity content of the MS-1 coating, resulting in the worst erosion resistance, which developed crack initiation sites on the cupola region of the coating, as seen in Figure 6d.

### 3.6 Thermo-Cyclic Durability of the Coatings

**Figure 7:**
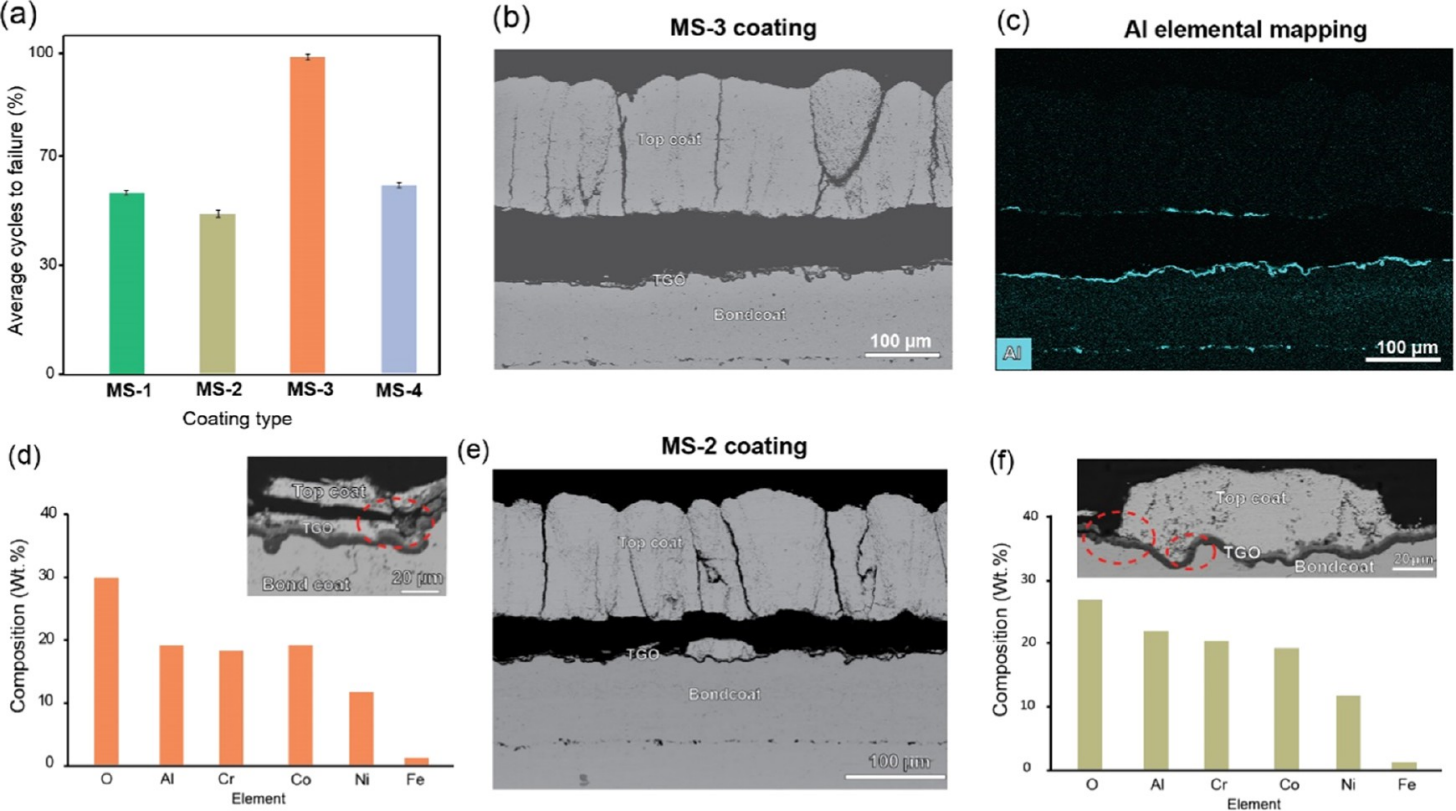
(a) Furnace cyclic endurance test of the coatings, (b) BSE image of the MS-3 coating post failure, (c) Al elemental energy-dispersive X-ray spectroscopy (EDS) map, (d) distribution of Spinel composition across the bond coat cross-section of the MS-3 coating, (e) BSE image of the MS-2 coating post failure, and (f) distribution of Spinel composition across the bond coat cross-section of the MS-2 coating.

The coatings were subjected to a furnace cycling test, and the results are shown in Figure 7a. The MS-1 and MS-4 coatings spalled at less than 60%, whereas the MS-2 coatings failed below 50%. All of the coatings exhibited a similar failure mechanism of complete spallation from the substrate; this phenomenon is most probably due to stress accumulation caused by the thickening of the TGO layer. TGO thickness of the MS-4 coating, as shown in Figure 7b, was 4.6 ± 0.3 μm, below the critical thickness of approximately 5–8 μm. Mahade et al. pointed out that TBC spallation occurs at a critical thickness of 5 μm during long thermal cyclic tests.^44,45^

The critical thickness is defined as the maximum thickness of incremental TGO growth before the occurrence of ultimate spallation of TBC. Vertical cracks or columns in both coatings widened, and some pre-existing lateral cracks propagated laterally. However, the top coat of both coatings remained intact without any evidence of lateral crack initiation or propagation at the interface of each layer. Energy-dispersive X-ray spectroscopy (EDX) elemental mapping of the MS-3 coating along the spalled cross-section in Figure 7c reveals the Al-rich TGO and indications of spinel formation at the interface between the top coat and bond coat from the composition presented in Figure 7d, as evidenced by the presence of Al, Cr, Ni, Co, and O elements in the EDX maps. The compositional percentage of elements suggests that remnants of the top coat material in the BSE image shown in Figure 7d were intact along the TGO layer, as corroborated by the backscattered electron (BSE) image of the same coating. MS-2 coating exhibited the least thermal cycling life, with a TGO thickness of 6.4 ± 0.5 μm, which falls within the critical thickness range. The spallation behavior was similar to MS-3 coating, as shown in Figure 7e, and the presence of Spinel oxides in MS-2 coating, with chromium oxide content marginally higher than MS-3 coating, as presented in Figure 7f. The incremental growth of the TGO results in a significant decline in fracture toughness and accumulation of internal stresses with each thermal cycle, as observed by Bolelli et al.^46^ In a comparative analysis between the most durable MS-3 coating and the less durable MS-2 coating, it was observed that both coatings exhibited microcrack formation originating from the thermally grown oxide (TGO)-bond coat interface, extending toward the coating. These observations are highlighted in the oval regions of Figure 7d,f. It is hypothesized that these microcracks significantly contributed to the spallation failure observed in the coatings. It should be noted that the MS-3 coating exhibited better thermal cycling resistance than the porous MS-4 coating; a similar observation was made by Zhou et al.^47^

### 3.7 In-Depth Study of Microstructure-Dependent CMAS Infiltration

Three coatings were downselected for the CMAS test; the MS-3 coating was chosen because of its superior thermal cycling resistance compared to other coatings, while the MS-4 coating was selected for its excellent erosion resistance and mechanical properties, and the MS-1 parameter was used as a point of reference for comparative purpose. CMAS attack on the coatings is multifaceted and involves a combinatory mode of failure. The cross-sectional BSE images and their corresponding overlapped Si elemental maps analyzed by EDX analysis of the MS-1 and MS-3 coatings are shown in Figures S7 and S8. From Figure 10a, it was observed that the MS-1 coating developed cracks after the infiltration of CMAS. The Si elemental overlapped image showed that the CMAS infiltrated into the interporosity bands and columnar gaps without any solidified residual CMAS on the top of the coating. This failure is mainly attributed to the culmination of the structurally weak properties discussed in the previous sections. CMAS completely infiltrated the MS-3 coating, which performed better in terms of thermal cycling, as evident from the greyish and dense regions in Figure S8a. The Si elemental map revealed that there was no residual solidified CMAS on the top layer. However, the columnar gaps and IPBs were completely infiltrated. It should be noted that the higher fracture toughness property made the MS-3 coating less prone to developing cracks in the coatings, as the CMAS solidified post infiltration into the coating.

**Figure 8:**
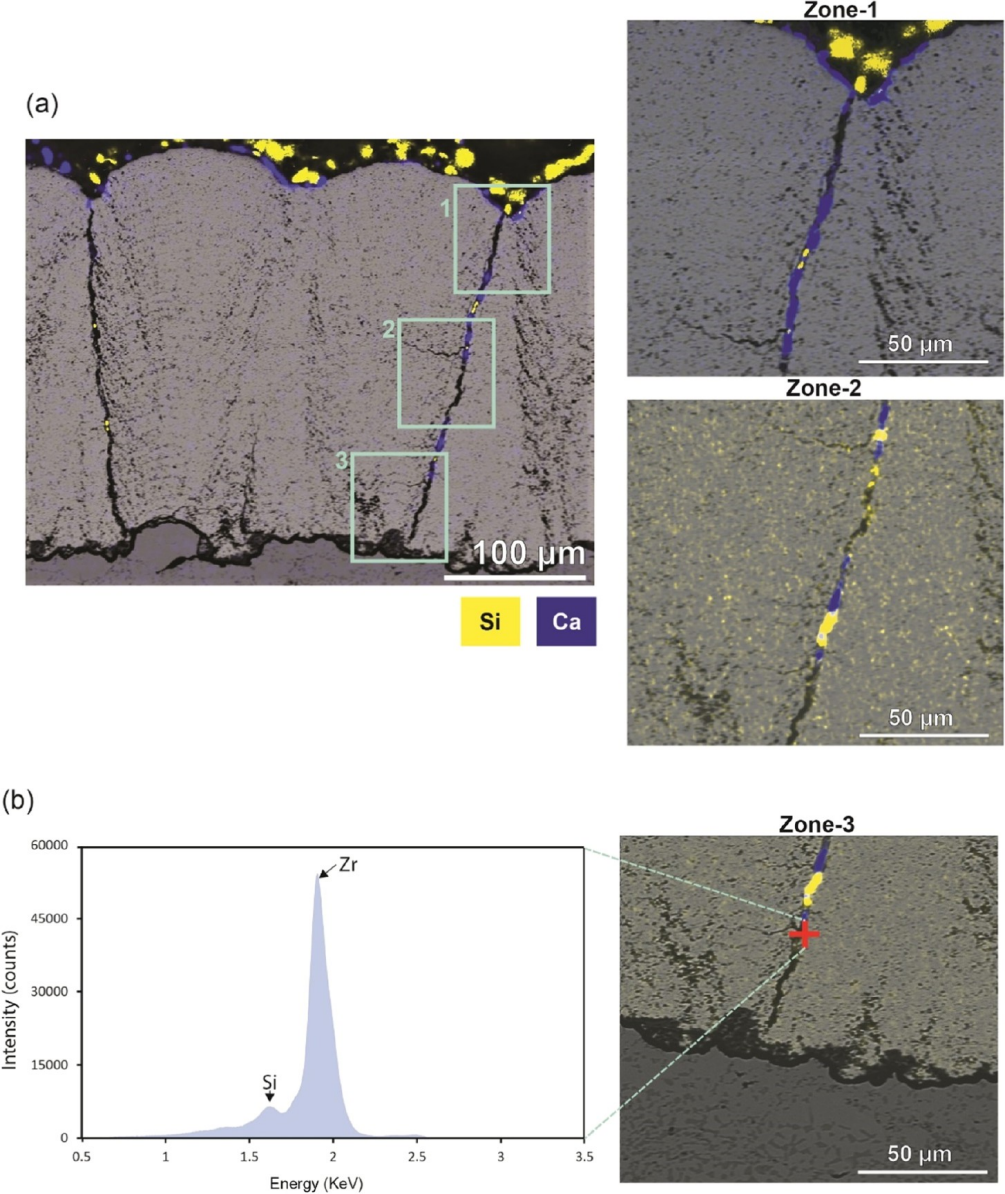
(a) Cross-sectional overlapped Si- and Ca-mapped BSE image and magnified regions tracing CMAS infiltration into the columnar crack of the MS-4 coating and (b) detecting small traces of Si in the bottom-most point of MS-4 coating.

The Si-overlapped image of the MS-4 coating revealed the presence of residual solidified CMAS in the coating. The surveillance of CMAS penetration into the columnar gaps is delineated across three distinct zones, revealing the existence of dendritic branching configurations. An in-depth look into the microstructural features of MS-4 post CMAS infiltration in Figure 8a revealed a lesser infiltration depth of 190 μm. The maximum infiltration depth of CMAS into the MS-4 coating was 276 μm in the columnar regions, which was confirmed by the elemental spectra of zone-3 in Figure 8b. Feathery cracks appeared to branch laterally from the columns, evident in Figure S9, which might decelerate the aggressiveness of CMAS; a similar phenomenon was published by Naraparaju et al. on developing the feathery columnar structured EB-PVD coatings.^37^ In-planar BSE images of the MS-1, MS-3, and MS-4 coatings ground and polished to a depth of 150 μm from the surface toward the substrate shown in Figure S10 revealed that the MS-1 coating had the highest intracolumnar porosity of 18.6% and a mean Feret diameter of 37 μm. The MS-3 had an intracolumnar porosity of 15.6% and a mean Feret diameter of 25 μm, and the MS-4 coating had an intracolumnar porosity of 10.3% and a mean Feret diameter of 19 μm. It is important to characterize the in-planar microstructural features, as they act as potential channels to accommodate the CMAS infiltration into the coatings. Such distinctive features shed light on the enhanced CMAS resilience exhibited by MS-4 coatings. The viscosity of CMAS at 1250 °C was estimated to be 1.4 Pas by using the Giordano silicate melt viscosity model^48^ (Figure S11). The surface tension of the molten CMAS was estimated to be 0.43 Pa·m using the Kucuk model.^49^

The infiltration of CMAS is predominantly propelled by capillary forces, representing a pivotal factor that governs the rate of penetration. Considering individual in-planar annular column as a capillary tube, the ensuing interaction between CMAS and the in-planar porous medium after wetting can be determined by evaluating the capillary pressure by employing the Young–Laplace equation.^50^
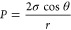
Where *P* is the capillary pressure, σ is the surface tension of the CMAS, θ is the static contact angle, and *r* is the effective radius of the pores. The average capillary pressure is presented in Figure S12a, with the MS-4 coating possessing the lowest capillary pressure of CMAS penetration with a value of 12.4 kPa, whereas MS-1 and MS-3 had capillary pressures of 14.6 and 14.8 kPa.

Figure S12b provides an intriguing inference, indicating that the MS-4 coating exhibits the greatest in-planar annular column area in comparison with the MS-1 and MS-3 coatings. When contemplating the CMAS infiltration tendencies of the MS-4 coating, it becomes evident that optimal circumstances for minimizing CMAS penetration into the coatings would entail a low in-planar porosity coupled with a high in-planar annular pore area. The permeability of the channels determines the aggressiveness of the CMAS infiltration into the coating, and it can be calculated using the Dvorkin equation,^51^ considering the columns as solid tube-like structures:
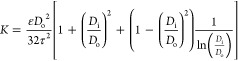
Where *K* is the permeability, ε is the porosity, τ is the tortuosity, *D*_o_ is the outer diameter of the in-planar columnar gap, and *D*_i_ is the in-planar column diameter. The MS-4 coating exhibited the lowest CMAS permeability value compared with MS-1 and MS-3 coatings (Figure S12c).

## 4 Discussion

### 4.1 Formation of Columnar Microstructures

The formation of columnar microstructures from a suspension feedstock relies on the creation of submicron-to-fine micrometric particles in the plasma jet and the factors that influence their deposition on the substrate. As shown in this work, the plasma and suspension conditions yielded conditions that form columnar coatings. The factors that govern the suspension and plasma properties are numerous and will not be discussed further here. This work primarily influences the coating deposition conditions at the substrate due to changes in the feed rate, stand-off distance, and robot speed. The formation of columns can be related to earlier work from Oberste-Berghaus et al.,^52^ in which the trajectory of fine particles (<5 μm) was shown to be influenced greatly by the flow of the plasma jet at the surface of the sample or component. Effectively, the fine in-flight particles produced by SPS are heavily influenced by the boundary layer at the substrate, reducing their normal velocity and increasing their velocity parallel to the substrate. VanEvery et al.^53^ further proposed that if coatings were deposited from a substantial proportion of particles having substantial velocity parallel to the substrate, then columnar microstructures would result. Fauchais et al.^18^ elaborated on this mathematically by looking at the Stokes number for small depositing particles in the boundary layer zone. Particles having a Stokes number of less than 1 will not penetrate through the boundary layer; those that have Stokes numbers greater than 1 will reach the substrate. The factors that influence the Stokes number are particle size, particle velocity, boundary layer thickness, and plasma viscosity. The deposition zone in an actual spray process is more complex because it contains zones with different particle conditions. Ganvir et al.^54^ performed a stationary deposition experiment that demonstrates the deposition from the zones practically. Larger particles with a high momentum are deposited from the core of the plasma with an almost perpendicular impact angle, resulting in “dense” structures. Smaller particles with less momentum are deposited off the centerline due to a stronger effect of the boundary layer, leading to a more oblique deposition trajectory. This deposition results in columnar features with more porosity.

This demonstrates that a coating that is deposited when the torch moves in and passes over the substrate will be built up from material from both the plasma core and periphery regions. Figure S13a,b demonstrates this difference in deposition trajectory as well as the different zones of deposition within the spray spot. In the case of the coatings studied in this work, the deposition conditions at the surface have been altered due to the changes in the experimental setup. For the coating sprayed with a reduced suspension feed rate (MS-2), the plasma is less loaded with material, resulting in higher particle velocities and temperatures. A reduced feed rate will also reduce the size of the deposition spot relative to the MS-1 condition. For the coatings at reduced stand-off distance (MS-3), the particles are, on average, arriving at the surface with a higher velocity and temperature relative to the MS-1 condition; thus, more particles will have a velocity high enough to overcome the boundary layer at the substrate (Stokes *N*_r_ > 1). The size of the core deposition zone is larger at the shorter stand-off. Local heat flux is also higher because the substrate is closer to the plasma jet. For the reduced robot surface speed (MS-4) conditions, the spray spot has a relatively longer dwell time over the substrate in comparison to the MS-1 condition. This results in thicker layers of material deposited from the plasma plume core and periphery zones, giving rise to clearer bands of interpass porosity in the microstructure. The higher dwell time also results in increased heat flux to the substrate from both the plasma and the depositing particles. Higher temperatures also lead to a tendency for more material to adhere and build up the coating. Higher temperatures impact the bonding of particles as well as mechanical properties such as hardness.

### 4.2 Spray Conditions for Improved Erosion and Furnace Cycling Performance

From the erosion test, it was observed that the mechanical properties of the coatings translated into erosion resistance, with the MS-4 coatings exhibiting the lowest erosion rates. Lima et al. conducted an extensive assessment of two variations of SPS coatings in contrast to a porous APS TBC and an EB-PVD coating.^55^ Notably, the SPS coatings displayed remarkable performance when exposed to similar conditions used in that study, surpassing the well-established standards dictated by the porosity levels of APS and EB-PVD coatings. It is widely acknowledged that coatings characterized by significant porosity levels tend to exhibit elevated erosion rates due to variations in hardness and fracture toughness.

**Figure 9:**
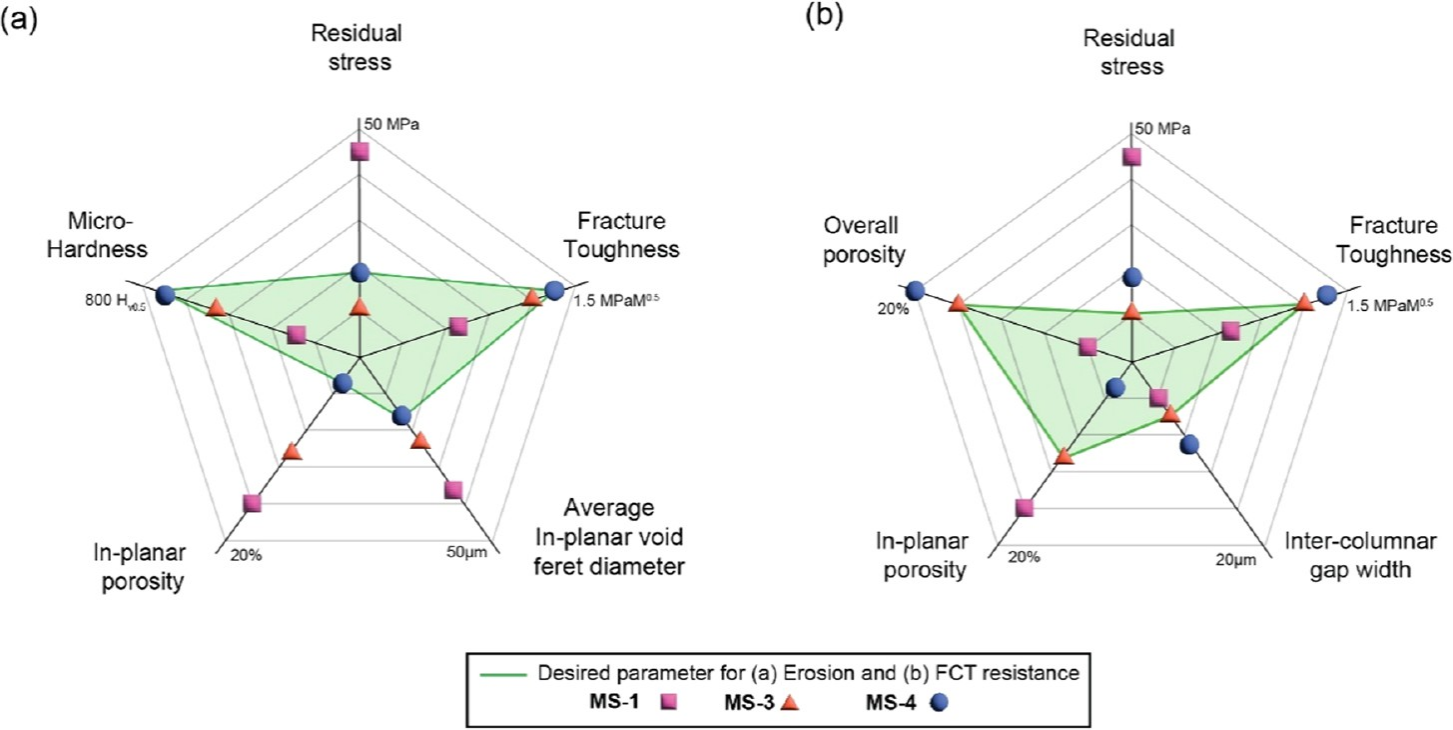
(a) Desired properties and microstructural features for erosion and (b) FCT resistance.

Figure 9a elucidates the key features and properties that underpin this resistance, while the geometrical aspects of the Feret diameter, a critical parameter in this context, are detailed in Figure S14. MS-2 coating was not included in the figure due to its lowest deposition rate and the lowest thermo-cyclic durability. Delving deeper into the pore size effects reveals that the average void Feret diameter in the MS-1 coating is substantially larger than that in the MS-4 coating. This difference in pore size is of significant interest because it correlates directly with the erosion behavior of these coatings. The MS-4 coatings, characterized by their minimal in-planar porosity, demonstrate superior hardness and fracture toughness. These attributes are intrinsically linked to the coating’s outstanding erosion resistance. Conversely, the larger pore sizes in the MS-1 coating imply a structurally less dense and potentially weaker matrix, which could be more susceptible to erosion. The MS-2 and MS-3 coatings, with their respective porosity and mechanical property profiles, exhibit a gradation in erosion resistance that falls between the extremes represented by MS-1 and MS-4. The mechanisms governing the behavior of these coatings under high-temperature thermal cycling include the exposure, physical attributes, growth of the TGO layer, mechanics within this layer, and the widening of cracks both vertically and horizontally due to the expansion and contraction of TBCs with each thermal cycle. The TGO layer’s formation is attributed to the diffusion of aluminum from the bond coat with the porous TBC oxide layer during high-temperature exposure. The outward diffusion of alumina in the early stages forms a slow-growing alumina layer (TGO layer). As the samples continued in the FCT, the Al composition throughout the bond coat thickness decreased, leading to a β-phase depletion. This change increases the oxygen activity at the TGO interface, resulting in more stresses in the top coat and eventual TBC failure. In a comparative analysis of deposition rates among MS-1, MS-4, and MS-3, it was observed that MS-1 exhibits a notably lower deposition rate. However, intriguingly, MS-1 manifests more than twice the residual stress compared to the other two. A salient distinction between the deposition processes of MS-3 and MS-4, as compared to MS-1, pertains to the heat input directed toward the substrate or coating during the deposition phase. This observation underscores the crucial significance of thoroughly examining the in-planar characteristics of coatings to accurately assess their durability for FCT, as shown in Figure 9b. MS-3 coating possessed the least in-plane residual stresses in comparison with the MS-4 coating, which resulted in the best thermal cycling resistance. Intriguingly, the MS-3 coating manifested an intermediate stance with respect to both cross-sectional and in-planar porosities. This lowest residual stress value, coupled with intermediate porosity values, is likely the reason for the better performance of this MS-3 microstructure. In contrast, the MS-1 and MS-4 coatings occupied the extremities of the porosity spectrum and had higher in-plane residual stresses, leading to poor cycling performance.

### 4.3 In-Planar Characterization of Columnar Microstructures Responsible for the CMAS Degradation

**Figure 10:**
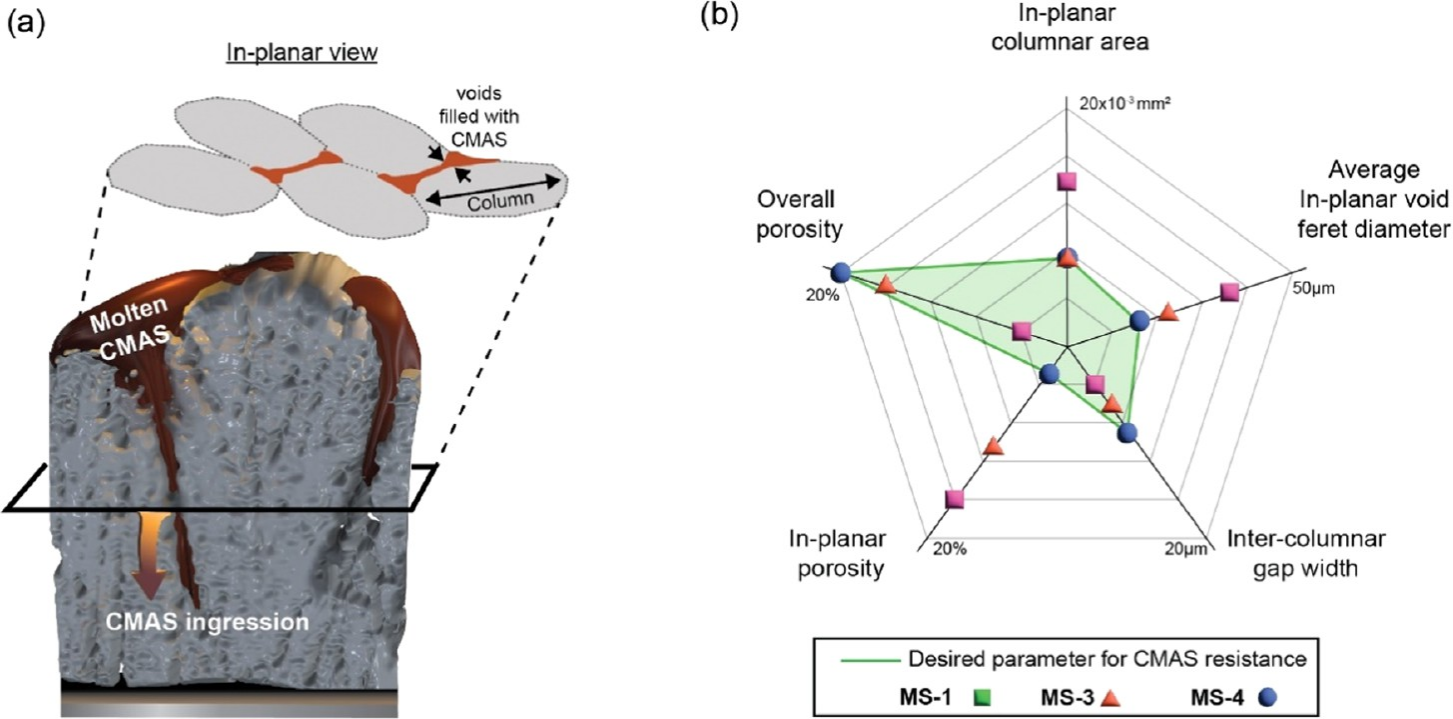
(a) Schematic illustration of the ingression of molten CMAS into the internal columnar gaps of the coating and (b) desired properties and microstructural features for CMAS resistance.

In this study, the CMAS infiltration was chosen for a short duration of five min at 1250 °C to understand the behavior and the implications of the microstructural features in their degradation. MS-4 coating possessed a dendritic branched microstructure in comparison with other coatings, leading to a distinct infiltration mechanism. The resistance to CMAS infiltration is a critical parameter that determines the resilience of thermal barrier coatings, which can be significantly enhanced by the thorough manipulation of the deposition conditions and engineering of their microstructure. The present investigation sets forth a deeper insight into the in-planar microstructural features that are crucial for engineering the coatings to offer CMAS resistance, as illustrated in Figure 10a and plotted in Figure 10b. The CMAS resistance exhibited by the MS-4 coating is majorly attributed to its lowest in-planar porosity and lowest in-planar void ferret diameter, which is also reflected in the lowest capillary pressure and capillary pressure compared to MS-1 and MS-3 coatings. The MS-4 and MS-3 coatings, as explored in this study, have showcased a remarkable balance of toughness and hardness, effectively preventing any potential for catastrophic cracking after the CMAS tests.

The MS-4 coating exhibited lower average in-plane residual stresses compared to the MS-3 coating, uniquely characterized by its dendritic branched formations and favorable in-planar columnar features, offering an augmented resistance to CMAS infiltration, a vital attribute for the durability of thermal barrier coatings.

## 5 Conclusions

This study undertook a thorough investigation to understand the spray parameters to develop columnar structures of Yttria-stabilized zirconia coatings produced by using suspension plasma spraying. In an effort to identify the most resilient coating, comprehensive thermo-chemico-mechanical characterization of coating variations was conducted, exploring the interdependent influences of the suspension feed rate (MS-2), stand-off distance (MS-3), and robot traverse speed (MS-4) parameters in relation to the reference (MS-1) parameter.

- Each of the coating variations displayed columnar microstructures, with MS-4 and MS-3 coatings exhibiting a greater deposition rate, leading to distinct interporosity bands and a similar overall range of porosity. The MS-4 coating stood out with the highest level of microhardness and fracture toughness compared to the other coatings.
- The MS-4 coating demonstrated remarkable resistance to erosion when compared to other variants, with the MS-1 coating displaying the lowest degree of resistance. This discrepancy in erosion performance can be ascribed to the disparities in the micromechanical properties.
- The MS-3 coating exhibited remarkable thermal cycling resistance, predominantly due to its inherent minimal residual stress and refined columnar microstructural features.
- The MS-4 coating exhibited a markedly enhanced performance in CMAS resistance, a distinction that is attributed to its intricate in-planar microstructural characteristics and the presence of dendritic microcrack segregations. The culmination of these microstructural features contributes to a comparatively lower CMAS permeability, bolstering its overall efficacy.

Through an endeavor to ascertain the optimal columnar microstructure with regard to thermo-chemico-mechanical robustness, the outcome led to the choice of MS-4 and MS-3 coatings, respectively, driven by their respective compatibility with the specified requirements. This study posits that the utilization of engineered SPS coatings exhibits the potential to supplant EB-PVD coatings and, concurrently, charts a course for the next frontier in thermo-chemico-mechanical durable coating development through optimization of the coating deposition parameters.

## Supporting Information Available

Backscattered electron (BSE) image and the particle size distribution of the YSZ particulates; XRD data of as-deposited SPS YSZ, and dried YSZ suspension powder; representative vickers microhardness indentations of MS-1, MS-2, MS-3, and MS-4 coatings; BSE image of the semimolten particles in the MS-4 coating; average in-plane residual stresses of the coatings; BSE image and the corresponding overlapped Si elemental map of the MS-1 coating post CMAS infiltration at 1250 °C for 10 min; BSE image, and the corresponding overlapped Si elemental map of the MS-3 coating post CMAS infiltration at 1250 °C for 10 min; BSE images of the overlapped Si elemental mapping at an infiltration depth of 190 μm, and the lateral branching of cracks of the MS-4 coating post CMAS infiltration at 1250 °C for 10 min; topographical BSE images of the as-deposited coating, and the frequency distribution of the pore ferret diameters of the MS-1, MS-3, and MS-4 coatings ground and polished to a depth of 150 μm toward the substrate; the change in viscosity of molten CMAS with respect to the temperature; average in-planar column area, capillary pressure of the CMAS infiltration, and permeability of the MS-1, MS-3, and MS-4 coatings; schematic of the trajectory of particles of different sizes, momentum in the plasma plume, and zones of deposition in the spray spot when looking directly down the center axis; schematic illustration of the geometric features examined in the coating (PDF)
