# Supplementary material for: Processing–Microstructure–Properties of Columns in Thermal Barrier Coatings: A Study of Thermo-Chemico-Mechanical Durability
Source: ACS Appl Mater Interfaces. 2024 Feb 13;16(8):10646–60. doi: 10.1021/acsami.3c16681 (PMC10910460; doi:10.1021/acsami.3c16681)
Supplement: Supplementary file 1 — am3c16681_si_001.pdf [file am3c16681_si_001.pdf]

SUPPORTING INFORMATION

Processing-microstructure-properties of columns in Thermal Barrier Coatings: A study of thermo-chemico-mechanical Durability

Siddharth Lokachari [a,c], Kah Leng [a], Acacio Rincon Romero [a], Nicholas Curry [d], Gyaneshwara Brewster [b], Andy Norton [b], Tanvir Hussain [a,c*]*

[a] Centre of Excellence in Coating and Surface Engineering, Faculty of Engineering, University of Nottingham, NG7 2RD

[b] Rolls-Royce plc, Derby, DE24 8BJ, UK

[c] Rolls-Royce UTC in Manufacturing and On-Wing Technology, Faculty of Engineering, University of Nottingham, NG7 2RD, UK

[d] Thermal spray innovations, Salzburg, Austria

Address correspondence to:

Siddharth.Lokachari@nottingham.ac.uk , Tanvir.Hussain@nottingham.ac.uk

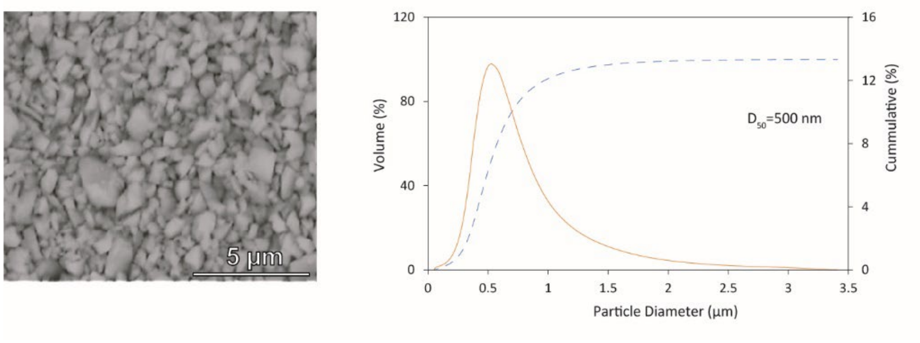

Figure S1. Backscattered Electron Image (BSE) and particle size of the YSZ particulates

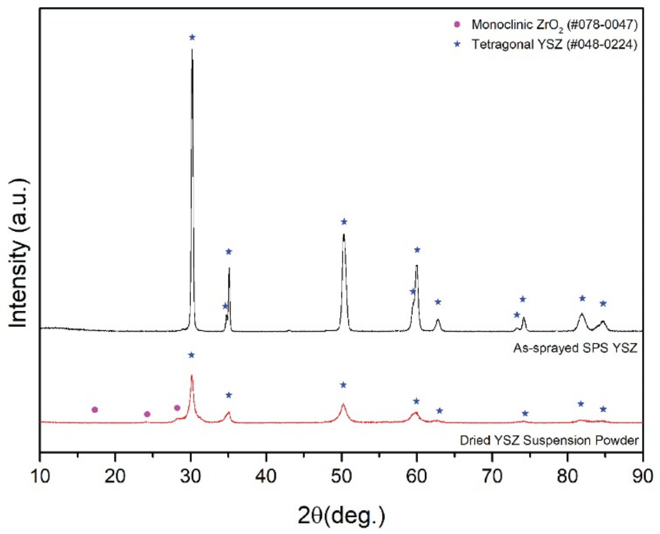

Figure S2. XRD data of As-deposited SPS YSZ and dried YSZ suspension powder

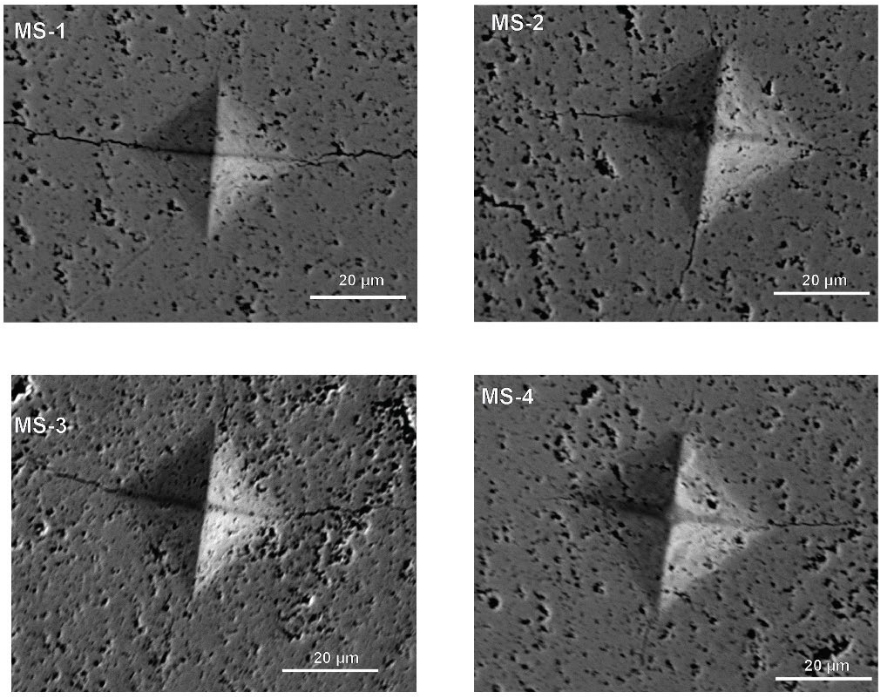

Figure S3. Representative Vickers micro-hardness indentations of MS-1, MS-2, MS-3 and MS-4 coatings.

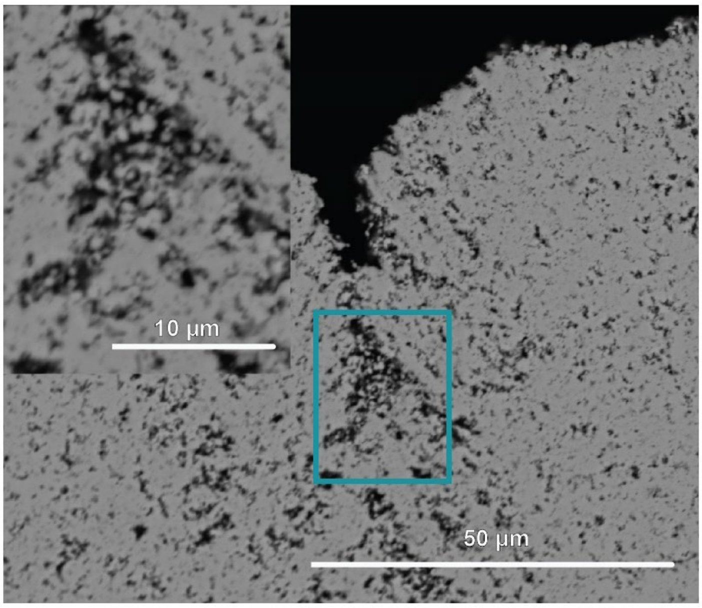

Figure S4. BSE image of the semi-molten particles in the MS-4 coating.

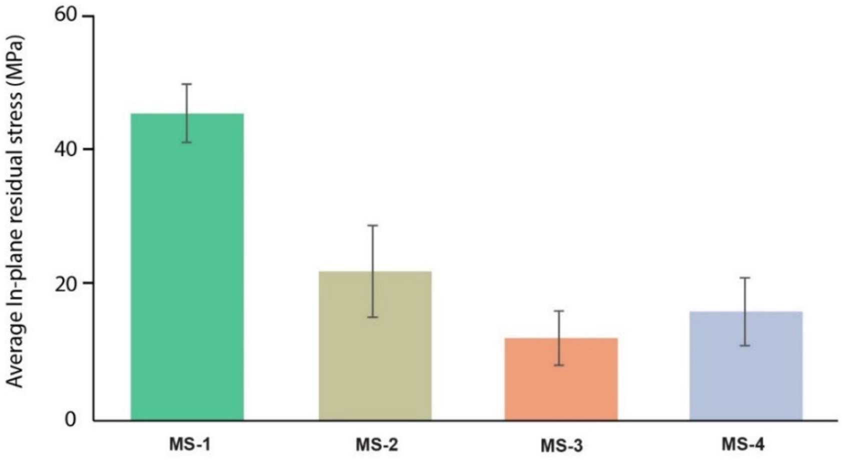

Figure S5. Average in-plane residual stresses of the coatings

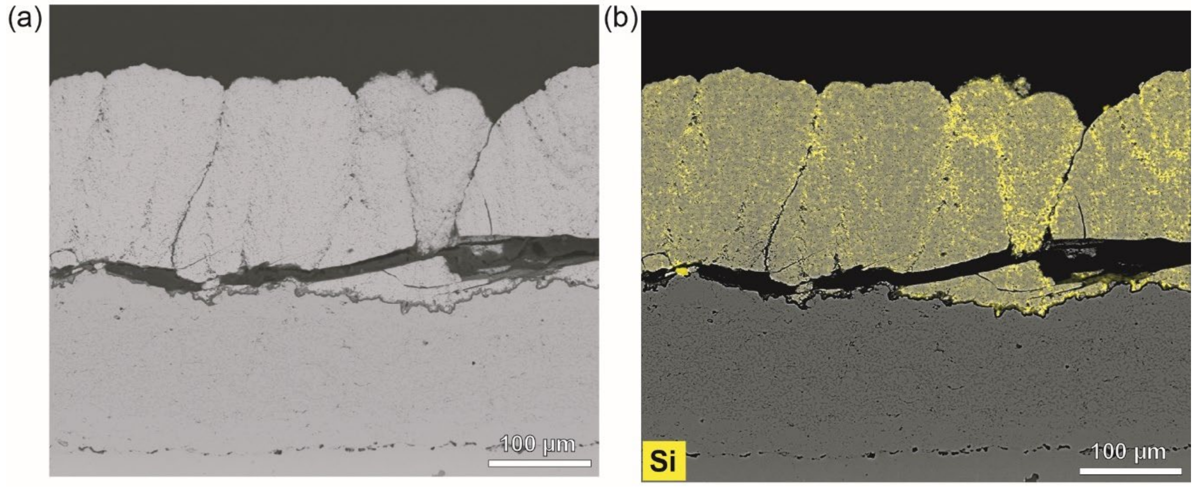

Figure S6. (a) BSE image, and (b) the corresponding overlapped Si elemental map of the MS-1 coating post CMAS infiltration at 1250°C for 10 minutes.

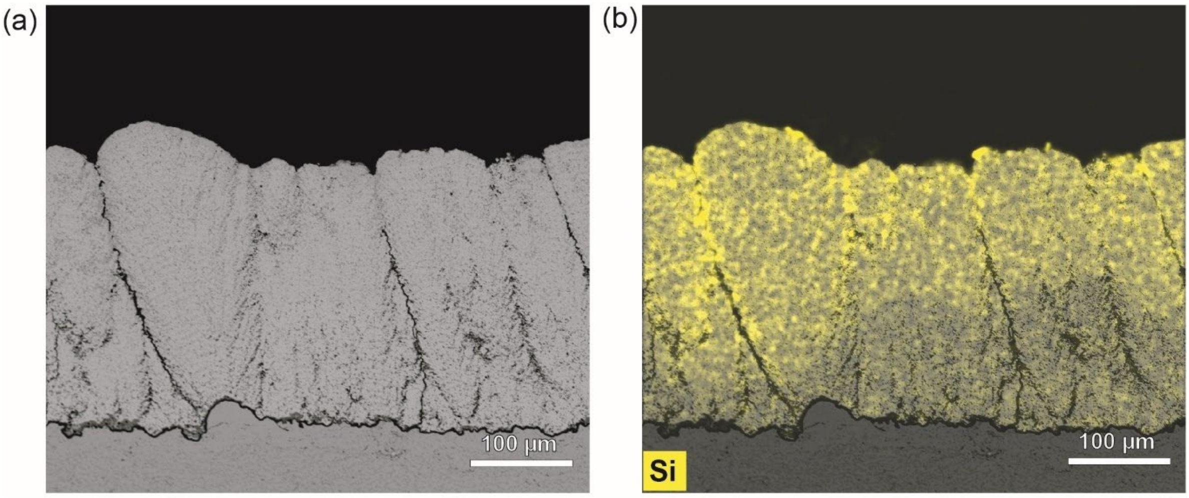

Figure S7. (a) BSE image, and (c) the corresponding overlapped Si elemental map of the MS-3 coating post CMAS infiltration at 1250°C for 10 minutes.

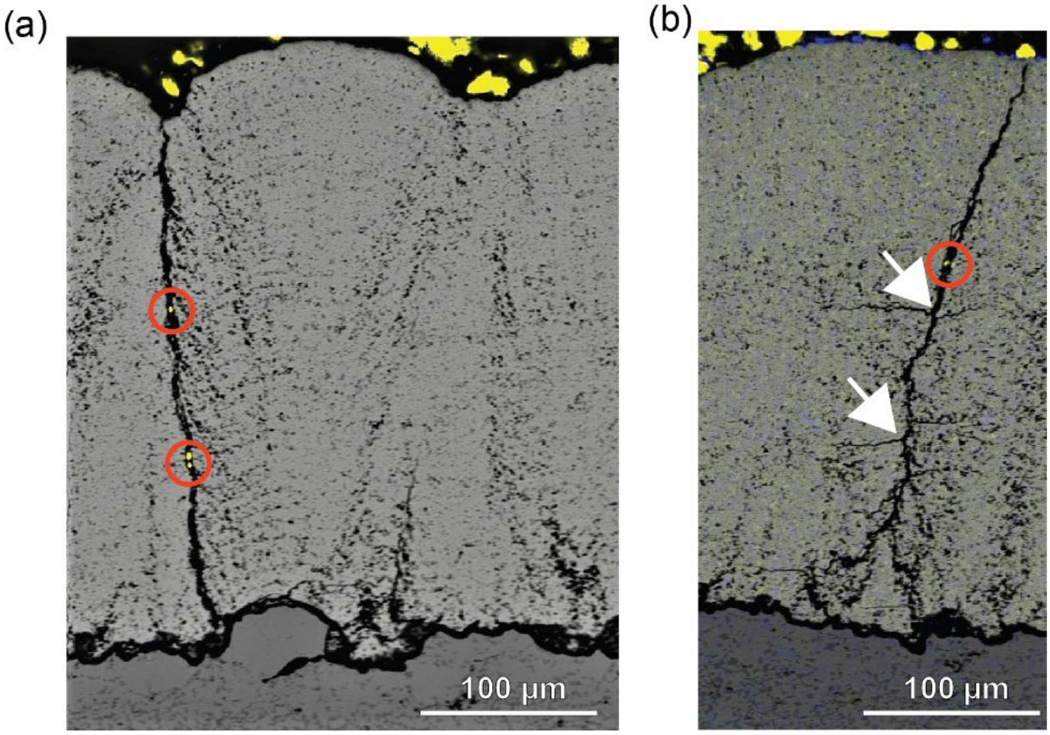

Figure S8. BSE images of the overlapped Si elemental mapping of (a) infiltration depth of 190 μm (b) the lateral branching of cracks of the MS-4 coating.

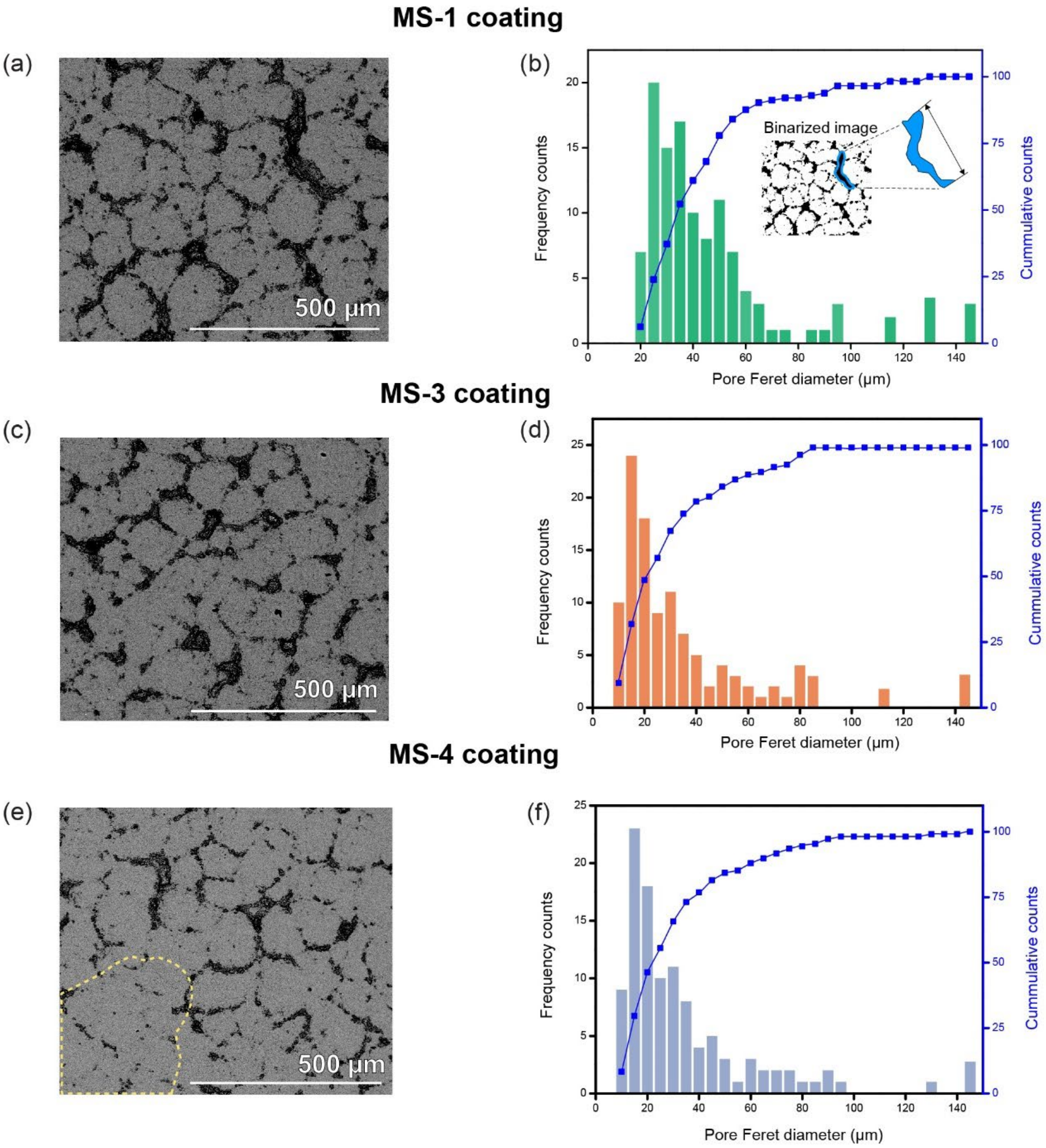

Figure S9. (a)Topographical BSE images of the as-deposited coating, (b) the frequency distribution of the pore ferret diameters of the MS-1 coating ground and polished to a depth of 150 μm towards the substrate, (c) topographical BSE images of the as-deposited MS-3 coating, (d) the frequency distribution of the pore ferret diameters of the MS-3 coating ground and polished to a depth of 150 μm towards the substrate, (e) topographical BSE images of the as-deposited MS-4 coating, and (f) the frequency distribution of the pore ferret diameters of the MS-4 coating ground and polished to a depth of 150 μm towards the substrate.

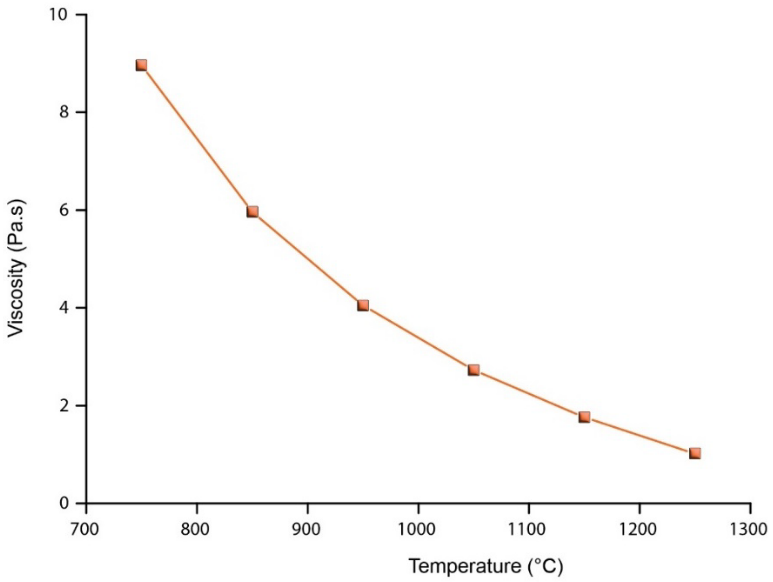

Figure S10. The Change in viscosity of molten CMAS with respect to the temperature

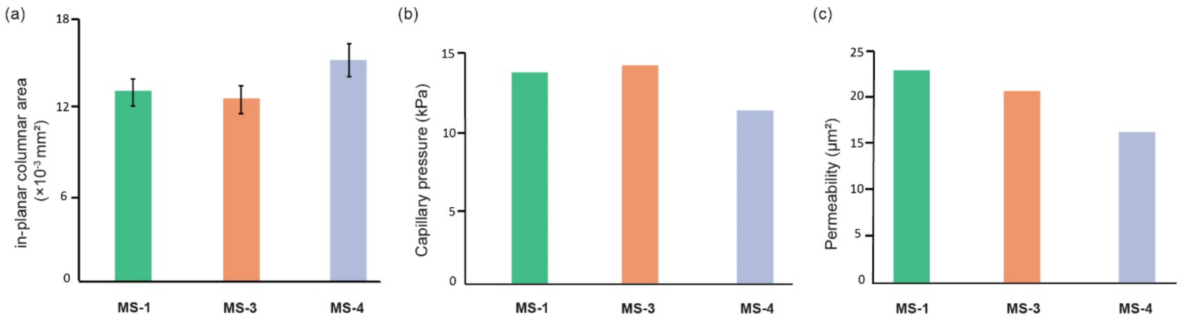

Figure S11. (a) Average in-planar column area, (b) capillary pressure of the CMAS infiltration, (c) permeability of the MS-1, MS-3 and MS-4 coatings.

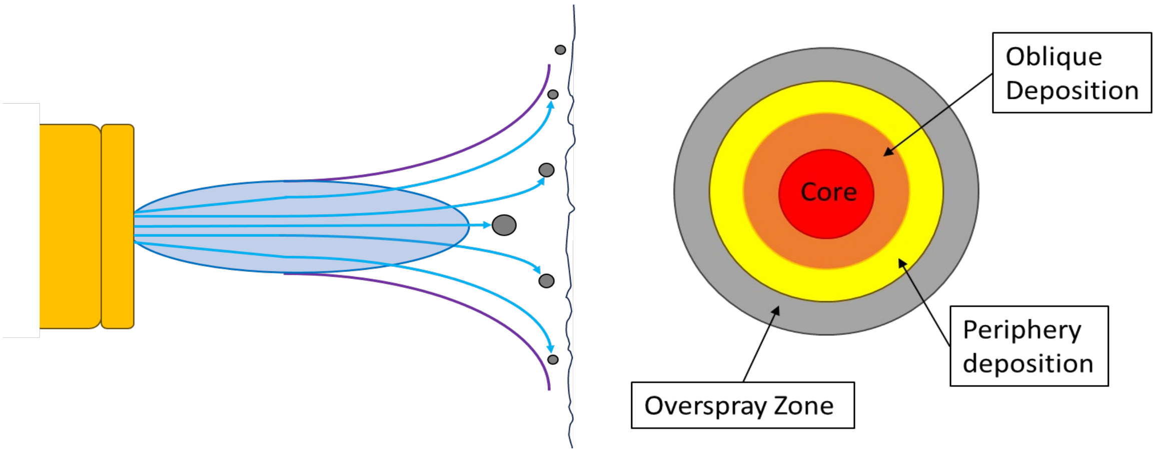

Figure S12. a) Schematic of the trajectory of particles of different sizes and momentum in the plasma plume, b) zones of deposition in the spray spot when looking directly down the center axis.

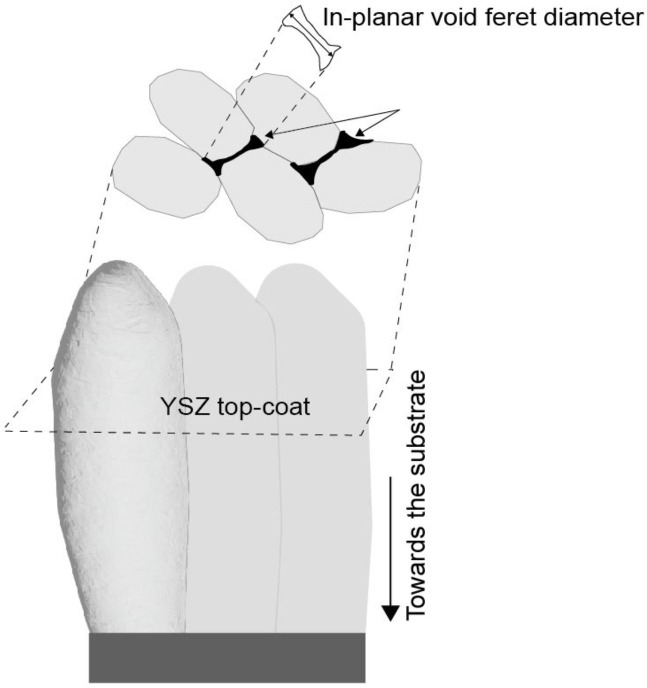

Figure S13. Schematic illustration of the geometric features examined in the coating.
